# Characterization of anti-leukemia components from *Indigo naturalis* using comprehensive two-dimensional K562/cell membrane chromatography and *in silico* target identification

**DOI:** 10.1038/srep25491

**Published:** 2016-05-06

**Authors:** Xunxun Wu, Xiaofei Chen, Jia Dan, Yan Cao, Shouhong Gao, Zhiying Guo, Philipp Zerbe, Yifeng Chai, Yong Diao, Lei Zhang

**Affiliations:** 1 School of Biomedical Science, Institute of Molecular Medicine, Huaqiao University, Quanzhou 362021, PR China; 2 School of Pharmacy, Shanghai Changzheng Hospital, Second Military Medical University, Shanghai 200433, PR China; 3 Department of Plant Biology, University of California, Davis, CA 95616, USA

## Abstract

Traditional Chinese Medicine (TCM) has been developed for thousands of years and has formed an integrated theoretical system based on a large amount of clinical practice. However, essential ingredients in TCM herbs have not been fully identified, and their precise mechanisms and targets are not elucidated. In this study, a new strategy combining comprehensive two-dimensional K562/cell membrane chromatographic system and *in silico* target identification was established to characterize active components from *Indigo naturalis*, a famous TCM herb that has been widely used for the treatment of leukemia in China, and their targets. Three active components, indirubin, tryptanthrin and isorhamnetin, were successfully characterized and their anti-leukemia effects were validated by cell viability and cell apoptosis assays. Isorhamnetin, with undefined cancer related targets, was selected for *in silico* target identification. Proto-oncogene tyrosine-protein kinase (Src) was identified as its membrane target and the dissociation constant (Kd) between Src and isorhamnetin was 3.81 μM. Furthermore, anti-leukemia effects of isorhamnetin were mediated by Src through inducing G2/M cell cycle arrest. The results demonstrated that the integrated strategy could efficiently characterize active components in TCM and their targets, which may bring a new light for a better understanding of the complex mechanism of herbal medicines.

Traditional Chinese Medicine (TCM), a complete system of healing developed in ancient China, is receiving more and more attention in China and throughout the world in recent decades^1^,^2^. However, the essential ingredients in TCM herbs have not been clearly identified and their precise mechanisms and targets have yet to be discovered, which seriously delays its integration into the modern health-care system^3^,^4^. How to characterize the active ingredients in TCM and their molecular targets is still the most challenging task at present^5^,^6^. Thus, the development of a new strategy is extremely critical to screen active ingredients and elucidate drug-target interactions^7^.

Traditionally, the components in TCM formulae were separated and identified by phytochemical methods, and evaluated by pharmacological assays for their molecular targets and mechanism, but the processes were incomprehensive, time-consuming and also inefficient^8^. In recent years, cell membrane chromatography (CMC) has been extensively used for active component screening and identification based on the interactions between membrane receptors and active ingredients^9^,^10^,^11^. It combines the advantages of both biomaterials and classic chromatography and realizes online and high-throughput screening of potential active ingredients from complicated biological samples^12^. In our previous study, a comprehensive two-dimensional (2D) high throughput screening system was firstly established which have successfully screened several active components from TCM herbs^8^,^13^. This biological chromatographic technique has been proven to be a powerful approach to screening active components from TCM.

*In silico* target identification, also known as reverse screening, is a technique that can be used to fast identify potential targets of small molecules and construct drug-target networks^14^. This approach has been successfully used to identify new potential biological targets for known compounds, and targets for compounds among a family of related receptors^15^,^16^,^17^. In recent years, a large number of computational target fishing methods and data bases have been developed^18^,^19^. In light of the existing huge amounts of components in TCM and their complex targets, this method may be a preferred strategy to explore the ingredient-target interaction and the functional mechanism underlying the multi-component combinations at the molecular level.

In this study, a new strategy that combines the comprehensive 2D K562/CMC system and *in silico* target identification has been developed to characterize active components and their targets in *Indigo naturalis* (Qingdai), an important TCM herb that has been used in several effective formulae for leukemia^20^,^21^. As shown in Fig. 1, a comprehensive 2D K562/CMC system was first established for screening potential active components from Qingdai. Then, the anti-leukemia effects of the screened components were verified by cell viability and apoptosis assays. Next, *in silico* target identification methods were employed for target screening. Compound-receptor interactions were further confirmed by molecular docking, CMC competitive displacement assays, kinase inhibition assays and surface plasmon resonance (SPR) analysis. Furthermore, receptor mediated molecular mechanism of K562 cell cycle regulation was analyzed. This novel methodology and strategy may provide a new way for characterizing active components from TCM and other complex systems and their targets.

## Results

### Identification of active components by comprehensive 2D K562/CMC system

**Figure 2:**
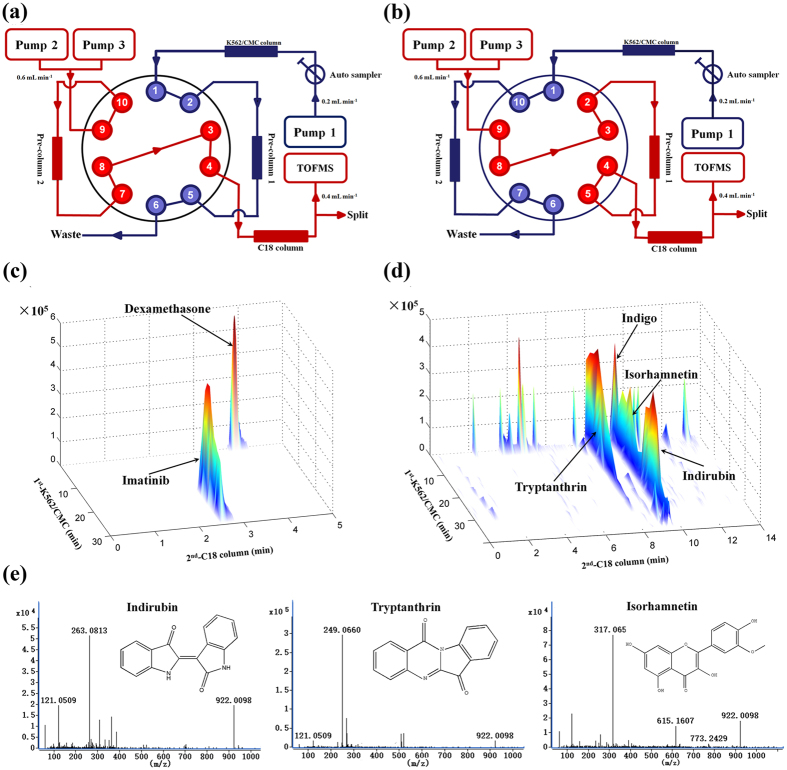
Construction and application of the proposed comprehensive 2D K562/CMC system Scheme of the 2D K562/CMC system. (**a**) K562/CMC column was equilibrated, and the 1^st^ fraction was collected in pre-column 1. (**b**) The 1^st^ fraction was analyzed by a C18 column coupled with TOFMS while the 2^nd^ fraction was collected in pre-column 2, then into the C18 column and TOFMS for analysis, alternately. (**c**) 3D plot of mixed standards obtained by 2D K562/CMC system. Dexamethasone was used as negative control, and showed no retention behavior on K562/CMC. Imatinib was used as positive control, and showed a significant retention characteristic on the K562/CMC. (**d**) 3D plot of Qingdai extracts obtained by 2D K562/CMC system. Three significant retention constituents were identified as indirubin, tryptanthrin and isorhamnetin, and one minor retention constituent was identified as indigo. (**e**) Chemical structures of potential bioactive components screened in Qingdai extracts.

A comprehensive two-dimensional K562/CMC system was first established based on our previous study^8^,^13^ (Fig. 2a,b). Two standard drugs, dexamethasone (binding to intracellular glucocorticoid receptor) and imatinib (acting on membrane receptor c-Kit and PDGFR) were selected to demonstrate the feasibility and selectivity of the proposed 2D K562/CMC system. The three-dimensional (3D) plot of mixed standards was shown in Fig. 2c. As expected, dexamethasone has minor retention behavior on K562/CMC system, while imatinib has a significant retention characteristic on the K562/CMC model.

Then, the comprehensive 2D K562**/**CMC system was used to screen anti-leukemia components from Qingdai extracts. As shown in Fig. 2d and Table 1, four membrane affinity components from Qingdai were identified according to our comprehensive identification of the 24 chemical components by quadruple-time-of-flight mass spectrometry (Q-TOFMS) (Supplementary Table S1). Then, a simultaneous quantitative determination of these four components was conducted in Qingdai. The contents of indigo, indirubin, tryptanthrin and isorhamnetin were 138.1 mg/g, 37.0 mg/g, 7.2 mg/g and 4.0 mg/g crude drug, respectively. At formula weight *m/z* 263.0813, there appeared two retention components (Fig. 2d). According to our previous results, one might be indigo, and the other might be indirubin. To further confirm our assumption, mixed standards of indirubin and indigo were selected for K562/CMC analysis (Supplementary Fig. S1). The results showed that the retention time of indigo on the 1^st^ dimension K562/CMC column was 2.5–7.5 min, while that of indirubin was 22.5–37.5 min. Thus, the three significant retention constituents were identified as indirubin, tryptanthrin and isorhamnetin, and the minor retention constituent was identified as indigo (Fig. 2e).

### Anti-leukemia effects validated by cell viability and cell apoptosis assays

**Figure 3:**
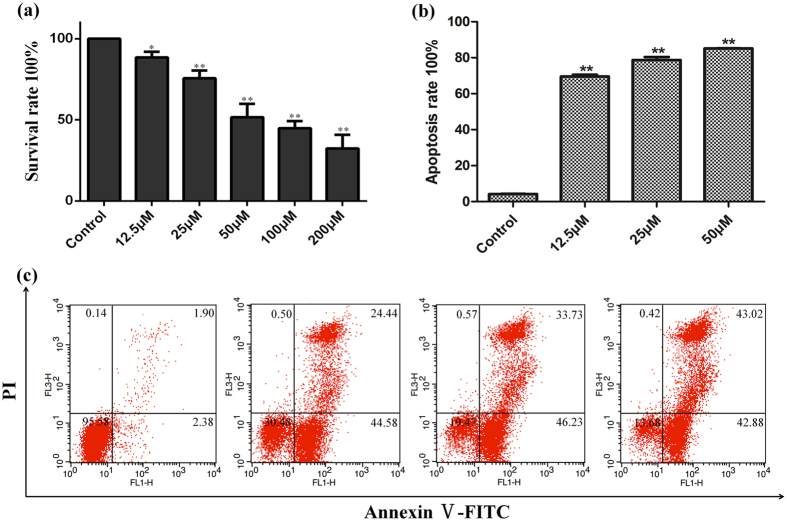
Cell vitality and apoptosis induced by isorhamnetin. (**a**) Effect of isorhamnetin on cell viability in K562 cells measured by CCK-8 assay after exposed to increased concentrations (0–200 μ M) for 48 h. (**b**) and (**c**) Cell apoptosis of isorhamnetin on K562 cells measured by Annexin V-FITC assay after exposed to increased concentrations (0–50 μ M) for 48 h. All data were shown as mean standard deviation (n =  6). Statistical significance was calculated by Student’s t-test.

In order to confirm the potential active components identified by the comprehensive 2D K562/CMC system, cell viability and cell apoptosis assays were adopted. The cell viability results of indirubin showed that the anti-leukemia effects occurred at a relative high concentration (100–200 μ M) (Supplementary Fig. S2). To be noted, because of its poor solubility in cell culture media, a large amount of this compound would be dissolved out^22^, the accurate anti-leukemia results of indirubin could not be achieved by cell viability assays *in vitro*. As for the other two components, isorhamnetin (Fig. 3) and tryptanthrin (Supplementary Fig. S3), they could also inhibit cell viability and induce apoptosis on K562 cells in a dose-dependent manner.

### Target identification for isorhamnetin

In this study, PharmMapper Server (http://lilab.ecust.edu.cn/pharmmapper/)^23^ and TargetHunter (http://www.cbligand.org/TargetHunter/)^24^ tools were selected for potential drug target identification. PharmMapper server is a spatial arrangement of features essential for a molecule to interact with a specific target receptor using pharmacophore mapping approach. By contrast, TargetHunter is built on biologically annotated chemical genomic (chemogenomic) databases with millions of bioactivity records, such as the ChEMBL database. These two methods, which based on different principles, are combinedly used in this study. Firstly, these two different tools are used for target screening (results shown in Supplementary Table S2 and Table S3). Then, the common targets obtained by these two methods were analyzed via Venny analysis (http://bioinfogp.cnb.csic.es/tools/venny/) (Supplementary Fig. S4). The receptors were ranked by fit score (Table 2). Estradiol 17-beta-dehydrogenase 1 is an enzyme that participates in androgen and estrogen metabolism, which seems to have no direct relation with leukemia. Aldose reductase is a cytosolic NADPH-dependent oxidoreductase that participates in glucose metabolism. It might be a receptor for isorhamnetin, but it didn’t appear at the cell membrane. However, Src is a non-receptor protein tyrosine kinase and could strongly interact with cellular membranes via an amino-terminal hydrophobic domain. The apparent Kd for binding of Src to the phosphatidyicholine/phosphatidylserine bilayer is 6 ×  10^−7^ M^24^,^25^. This interaction is sufficiently strong to account for Src membrane targeting^25^,^26^. It is also a very important therapeutic target for leukemia in clinical practice, such as dasatinib, a widely used drug targeting at Src^27^. Thus, Src was selected for further validation.

### Validation of isorhamnetin binding to Src

**Figure 4:**
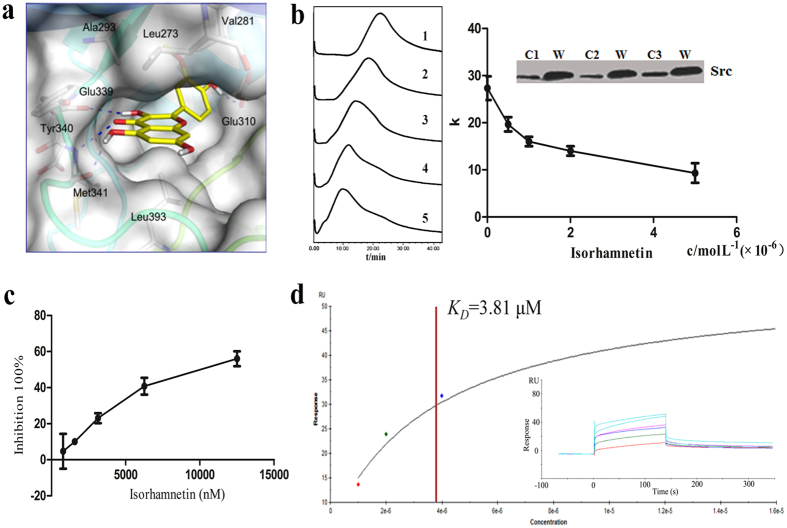
Validation of isorhamnetin binding to Src kinase (**a**) Isorhamnetin docked in the ATP binding site of the human Src kinase domain. Relevant hydrogen bonds are shown in blue dashed lines. (**b**) Left panel: Chromatogram of dasatinib (10 mM, 5 μ L) on the K562/CMC system using 10 mM ammonium acetate buffer as the mobile phase. Mobile phases were added with (1) 0 μ mol/L; (2) 0.5 μ mol/L; (3) 1 μ mol/L ; (4) 2 μ mol/L; (5) 5 μ mol/L isorhamnetin, respectively. Right panel: Competitive displacement assay of isorhamnetin on K562/CMC columns (presented as *k* values) with different isorhamnetin concentrations (0, 0.5, 1, 2, and 5 μ M) added to the mobile phase. Protein levels of Src proteins in K562/CMC columns (C1, C2 and C3) and whole cells (W). (**c**) Selective ATP-competitive kinase assay of isorhamnetin through caliper mobility shift assay. (**d**) Representative example of isorhamnetin binding to Src kinase by SPR. Immobilized on Series S sensor chip CM5, and then the indicated concentrations of isorhamnetin were injected over the chip to obtain the sensorgrams. The 1:1 binding fitting model was used to determine the kinetic parameters. Error bars represent the standard deviation based on three independent samples (n =  3).

*In silico* target identification results suggested that Src kinase might be a receptor of isorhamnetin. To further validate that isorhamnetin could strongly interact with Src kinase on cellular membrane, different methods were selected to verify the interaction. Firstly, molecular docking study^28^ was selected and results showed that isorhamnetin was able to form three hydrogen bonds with the so-called hinge region, the sequence segment that connects the N-lobe to the C-lobe of the kinase domain (Fig. 4a). Hydrogen bonding with the hinge region is one feature of type I kinase inhibitors. The core rings of both compounds are further involved into a lipophilic sandwich interaction between Leu273, Val281, Ala293 and Tyr340 on one side, and Leu393 on the other side of the catalytic cleft. Notably, the binding of isorhamnetin extends into the back pocket of the ATP-site with its methoxyphenyl motif in an orientation that is very similar to bosutinib, and it forms one additional hydrogen bond with the catalytic important Glu310 by the hydroxyl group.

Next, several K562/CMC columns were randomly selected and the existence of Src on cell membrane was verified by western-blotting assay (Fig. 4b). CMC competitive displacement assay was adopted using an existing Src ligand, dasatinib. Firstly, different concentrations of isorhamnetin (0 μ mol/L, 0.5 μ mol/L, 1 μ mol/L, 2 μ mol/L, 5 μ mol/L, respectively) were added to the mobile phase as a competitor. The retention time of dasatinib decreased in the K562/CMC model with an increasing isorhamnetin concentration (Fig. 4b). This result indicated that dasatinib competed with the same receptor with isorhamnetin.

In order to test the effect of isorhamnetin on the activity of Src kinase, the *in vitro* kinase inhibitory assay was performed^29^. The results showed that the IC_50_ of isorhamnetin was 6.9 μ M (Fig. 4c). SPR analysis was selected to further test the direct binding of isorhamnetin to Src. The results showed that the Kd between isorhamnetin and Src was 3.81 μ M (Fig. 4d).

### Anti-leukemia mechanism of isorhamnetin

**Figure 5:**
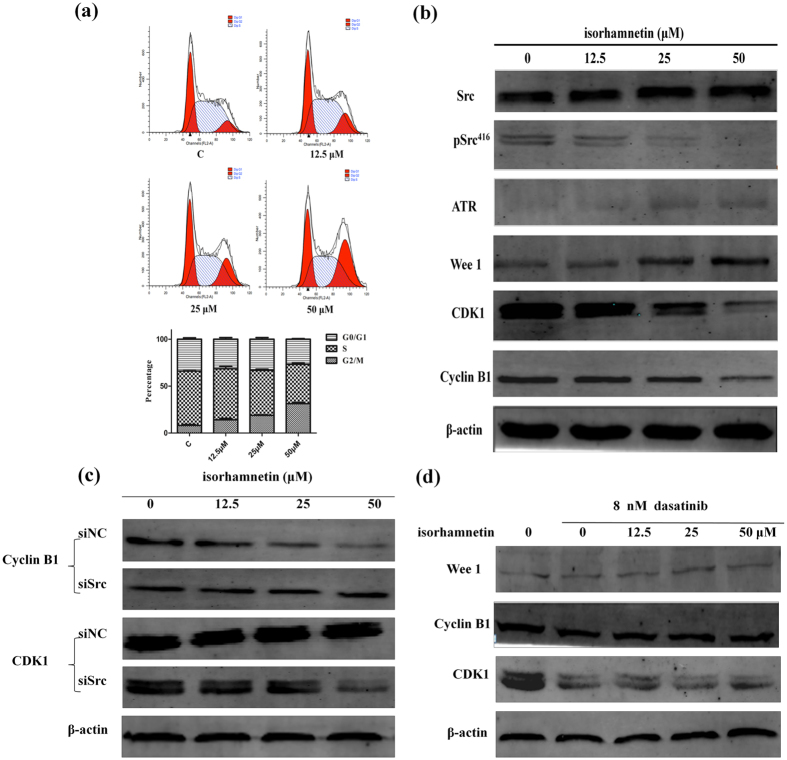
Isorhamnetin arrests K562 cells at G2/M phase via Src. (**a**) The effects of isorhamnetin on cell-cycle progression determined by flow cytometry. Representative cell-cycle distributions after exposure to 0, 12.5, 25 and 50 μ M isorhamnetin for 24 h. (**b**) Western blotting analysis of whole cell extracts of K562 cells treated for 24 hours with different doses of isorhamnetin, showing the dose-dependent down-regulation of pSrc^416^, CDK1, Cyclin B1 and up-regulation of ATR and Wee 1. pSrc^416^ indicates the phosphoryl-specific antibody targeting activated Src phosphorylated at Y416. (**c**) K562 cells were transfected with Src siRNA or negative control siRNA for 48 hours, then K562 cells were treated with different doses of isorhamnetin. Western blotting analysis of whole cell extracts showed the dose-dependent down-regulation of Cyclin B1 and CDK1 in negative control group. These two proteins displayed no remarkable change in Src siRNA group. (**d**) The K562 cells were pre-treated with 8 nM dasatinib (the first line is control) for 12 h, then the K562 cells were treated with different doses of isorhamnetin for 24 h. The protein levels are shown in the above picture. (The typical full blot of the western blot is shown in Supplementary material).

To further understand the underlying mechanisms of the anti- leukemia effects of isorhamnetin, its influence on K562 cell cycle was then analyzed. The data indicated that during the 24 h time period, isorhamnetin increased the percentage of G2/M phases in a dose-dependent manner (Fig. 5a). Then, the cell cycle checkpoint proteins were analyzed by western-blotting assay. The results demonstrated that the Src relevant pathway molecules such as pSrc, CDK1, Cyclin B1 were down-regulated, while ATR, Wee1 were up-regulated by isorhamnetin with a dose-dependent manner (Fig. 5b). To further verify that the effects on cell cycle regulation of isorhamnetin were through ATR, caffeine (an ATR inhibitor) was used to treat K562 cells for 0 h, 1 h, 3 h, 6 h and 12 h. The results demonstrated caffeine could almost completely neutralize the effects of isorhamnetin (Supplementary Fig. S5). These results demonstrated that isorhamnetin-induced cell cycle arrest was partly through Src/ATR/Wee 1/Cyclin B1/CDK1 pathway.

In order to demonstrate that the cell cycle arrest at G2/M phase induced by isorhamnetin was directly through Src, the Src receptor was knocked down by Src siRNA or the phosphorylation state of Src was suppressed by dasatinib. Firstly, the most effective siRNA and suitable concentration of dasatinib were screened. According to the screened results (Supplementary Figs S6 and S8 and Table S4), Src siRNA-2, negative control (NC) siRNA and dasatinib at 8 nM were selected for further study. Then, the cell cycle was assayed by flow cytometry and the cell cycle checkpoint related proteins were analyzed by western blotting assays. After the Src receptor was knocked down, isorhamnetin could not arrest cell cycle at G2/M phase anymore in a dose-dependent manner (Supplementary Fig. S7). It had minor effects or even no effect on cell cycle regulation. According to the western-blotting results, transfection of K562 cells with Src siRNA also reversed the isorhamnetin inhibition of Cyclin B1 and CDK1 activation seen in cells transfected with the NC siRNA (Fig. 5c), indicating that activation of the Cyclin B1 and CDK1 pathways were in part regulated by Src in K562 cells. In addition, after the phosphorylation state of Src^416^ was suppressed by dasatinib, the cell cycle regulation effects of isorhamnetin have also been arrested (Supplementary Fig. S9). The western-blotting results showed that the protein levels of Wee 1, Cyclin B1 and CDK1 had no significant difference after treatment of isorhamnetin at different concentrations (Fig. 5d), indicating that the regulation of the Wee 1, Cyclin B1 and CDK1 pathways was in part through the phosphorylation of Src^416^.

## Discussion

In view of the vast amounts of TCM formulae and components, only a small portion of active components and targets have been validated before. The lack of efficient strategies and methodologies limits the modernization of TCM. On the basis of our prior research, an integrated strategy was established for the characterization of anti-leukemia components from *Indigo naturalis* using comprehensive 2D K562/CMC and *in silico* target identification.

CMC is an affinity chromatography technology which is based on the interaction between active components and membrane receptors. It has been widely used for screening and identification of active molecules in complex samples. The advantage of CMC is that it is widely applicable to any complex samples including TCM herbs without requiring isolation or purification of individual small molecules. Moreover, the method is a comprehensive and efficient approach for active ingredient screening and identification. The cell membranes can be obtained from any cells or tissues in physiological or pathological states. When identifying a specific target-binding molecule, an overexpression or reduced expression state membrane receptor can also be used^30^,^31^. In our previous study, a novel comprehensive 2D CMC system was established^8^ and several active components were successfully identified via that system. In this study, a sensitive comprehensive 2D K562/CMC system combined with optimized stable columns^9^ was first established for rapid screening and characterization of potential active components from Qingdai. In comparison with the previous CMC online systems, the optimized columns were first applied to the comprehensive 2D K562/CMC system and two pre-columns were adopted to improve the sensitivity. Qingdai is a famous TCM herb and is widely used as a therapeutic agent for leukemia in China^20^,^21^. The published active component in Qingdai was mainly about indirubin. However, clinical and preclinical experiments showed that despite its anti-leukemia effects, its poor solubility in water and severe side-effects limit its clinical use^22^,^32^. This demonstrated that the satisfactory clinical curative effects of Qingdai might rely on other active components. Thus, characterization of the anti-leukemia components in Qingdai and their targets is a big task ahead of us.

Using the 2D K562/CMC system, three active components were screened. In this study, the anti-leukemia effects of indirubin have also been verified. To be noted, because of its poor water solubility, a large amount of this compound would be dissolved out^22^, so the accurate anti-leukemia results of indirubin could not be achieved by cell viability assays *in vitro*. Since the poor solubility in water and severe side-effects limit the clinical use of indirubin, lots of indirubin derivatives with a high efficacy and low toxicity have been developed^33^,^34^. If the water solubility of indirubin could be improved such as formulation in self-emulsifying drug delivery systems containing E804 (indirubin-3′ -oxime 2,3-dihydroxypropyl ether) and 5-OH-5-nitro-Indirubin oxime (AGM130), it would have much better anti-leukemia effects^35^,^36^. In a word, indirubin is not a satisfying drug, but it could be a good pro-drug. Fortunately, the other two components, tryptanthrin and isorhamnetin, both showed satisfactory anti-leukemia effects, and the results were in accordance with the previous study. Previous research reported that tryptanthrin could inhibit cell viability and induce apoptosis in leukemia^37^ and other cancer cells including breast cancer^38^ and lung cancer^39^. One of the direct cancer related targets of tryptanthrin was verified as cyclooxygenase-2^40^. However, the cancer-related targets of isorhamnetin have been rarely reported before.

Target identification of the known active compounds is an important research field in the modernization of TCM. It is also a challenging and costly step towards chemical biology and phenotypic screening^41^. *In silico* identification of potential biological targets for bioactive compounds offers an alternative avenue for the exploration of ligand–target interactions and biochemical mechanisms. *In silico* target screening methods are employed to identify the receptors of isorhamnetin. These methods are designed to identify the most probable target of a query molecule and different methods are always based on different principles, such as chemical similarity searching, data mining/machine learning, panel docking and the bioactivity spectral analysis^18^. Compared with the experimental methods, it is more efficient, less costly and easier to perform^42^. What’s more, the complex composition of TCM makes it even harder to elucidate the multi-target mode of action between compounds and targets via experimental methods. Here, we adopted *in silico* identification methods and identified Src as a direct receptor of isorhamnetin. Previous studies reported that isorhamnetin induced C-terminal Src kinase expression and inhibited Src activation in colorectal cancer cells, but it didn’t bind directly to Src^43^. In this study, various methods were adopted to verify the interaction between Src receptor and isorhamnetin. Molecular docking study, CMC competitive experiments, SPR analysis and *in vitro* kinase inhibition assay results all indicated that Src was a direct target of isorhamnetin.

Cell growth and proliferation are mediated via cell cycle progression and regulation of the cell cycle has become a novel target for the management of cancer. The cell cycle is a critical regulator of the processes of cell proliferation and growth after DNA damage^44^,^45^. Previous studies^45^,^46^ suggested that the anti-leukemia effects of isorhamnetin might be through inducing DNA damage. In order to explore the molecular mechanism of isorhamnetin via Src, cell cycle analysis was performed and the results showed that isorhamnetin could arrest K562 cells at G2/M phase. Western blotting results showed a dose-dependent inhibition of Src^416^ phosphorylation by isorhamnetin. Cell cycle related proteins, such as ATR and Wee 1, were up-regulated, while CDK1 and cyclinB1 were down-regulated with the presence of isorhamnetin. The CDK1/cyclin B1 complex has been well known as the regulators governing the G2 to M phase progression or inducing apoptosis^47^,^48^,^49^. The results demonstrated that the cell cycle regulation effects of isorhamnetin were through ATR/Wee 1/Cyclin B1/CDK1 pathways.

To determine if the effects of isorhamnetin on cell cycle regulation were directly through Src receptor, the Src receptor was knocked down with siRNA or the phosphorylation state of Src was suppressed by dasatinib. Transfection of K562 cells with Src siRNA also reversed the isorhamnetin inhibition of Cyclin B1 and CDK1 activation seen in cells transfected with the NC siRNA, indicating that activation of the Cyclin B1 and CDK1 pathways was in part regulated by Src in K562 cells. Then, after the phosphorylation state of Src^416^ was suppressed by dasatinib, the protein levels of Wee 1, Cyclin B1 and CDK1 had no significant difference after treatment with isorhamnetin at different concentrations, indicating that the regulation of the Wee 1, Cyclin B1 and CDK1 pathways was in part through the phosphorylation of Src^416^. Taken together, the anti-leukemia effects of isorhamnetin were directly through Src by arresting leukemia cells at G2/M and then inducing cell apoptosis.

In conclusion, we have established an integrated strategy of CMC-based active component screening and *in silico* identification to efficiently elucidate the active components and find their potential molecular targets. Three active components were successfully screened and Src was verified as a direct target of isorhamnetin, suggesting that isorhamnetin might be a new potent chemopreventive drug candidate or a lead compound for the treatment of leukemia. This efficient, practical, and universally applicable strategy might also be used for the identification of active molecules and their targets in other complex samples besides TCMs, such as drug-containing serum, metabolites or mixtures of synthetic compounds.

## Methods

### Reagents and chemicals

Authentic standards of higher than 99.0% purity were purchased from the following sources: imatinib and dexamethasone, Nanjing Ange Pharmaceutical Co. Ltd. (Nanjing, China); indirubin and indigo, Aladdin^®^ (Shanghai, China); dasatinib, Dalian Meilun Biology Technology Co. Ltd (Dalian China); Qingdai (collection in Fujian, China) Shanghai Leiyunshang Medicine Corp. (Shanghai, China). Other chemicals and reagents: isorhamnetin, Sigma-Aldrich (USA); cell counting kit-8 (CCK-8), Dojindo Molecular Technologies (Dojindo Molecular Technologies, Japan); annexin V-FITC apoptosis detection kit, BD PharmingenTM, BD Biosciences; RIPA (Radio Immunoprecipitation Assay) lysis buffer, Beyotime Co. (Jiangsu, China). Antibodies against Src, pSrc^416^, cyclin B, ATR, Wee 1 and β -actin, Cell Signaling Technology (USA); DyLight^TM^ 680-labeled antibody to rabbit IgG (H+ L), KPL Inc. (Gaithersburg, MD, USA); CDK1, (Abcam laboratories, Cambridge, MA, USA). Other reagents were of analytical grade.

### Sample preparation

Standard solutions of dexamethasone (10 mM) were prepared in methanol and imatinib was prepared in ultrapure water (pH 2.5). Isorhamnetin and dasatinib for cell viability assays were prepared in DMSO (20 mM) and diluted to different concentrations with complete RPMI-1640. The end concentration of DMSO was < 0.1%. Qingdai (2.5 g) was extracted with 25 ml CHCl_3_ (with 2% chloral hydrate) for 60 min using an ultrasonic extractor. The resulting extract was centrifuged at 12000 ×  g for 5 min and the supernatant filtered through 0.22 μ M nylon filters.

### Cell culture

The K562 cell line was obtained from the American Type Culture Collection (Manassas, VA). Cells were cultured in RPMI-1640 medium containing 10% FBS, 100 U/ml penicillin and 100 μ g/ml streptomycin at 37 °C and in a humidified CO_2_ (5%) atmosphere.

### Construction of the proposed comprehensive 2D K562/CMC system

K562/CMC column (10 ×  2 mm i.d., 5 μ m) preparation, cell viability assay, and CMC competitive displacement assay were performed according to the previously reported method^8^,^9^,^13^. Comprehensive 2D K562/CMC system was constructed according to our previously reported method^8^ with some modifications. K562/CMC column was applied as the first dimensional column. Two 500 μ L sampling loops were replaced by two XDB-C18 pre-columns (12.5 ×  4.6 mm i.d., 5 μ m, Agilent). For the second dimension separation, an XBridge^TM^ C18 column (100 ×  3.0 mm i.d., 3.5 μ m, Waters, Ireland) was used and the flow rate was 0.6 mL/min. Chromatographic and mass spectrometer conditions are shown in Supplementary methods.

### Cell apoptosis assay

The Annexin V-FITC apoptosis detection kit I was used to detect and quantify apoptosis by flow cytometry according to the manufacturer’s protocol. In brief, treated and untreated K562 cells were prepared as follows: A total of 5 ×  10^5^ cells were collected, washed with PBS (10 mM, pH 7.4), and mixed with 195 μ l of Annexin V-FITC binding buffer and 5 μ l of Annexin V-FITC (under gently mixing in this order). Next, 10 μ l of propidium iodide (PI) were added to the cells and incubated for 15 min at room temperature in the dark. Cell apoptosis was immediately analyzed using BD CellQuest Pro software by FACS Calibur.

### Cell cycle assay

K562 cells were harvested and washed with cold PBS (10 mM, pH 7.4) by centrifugation at 1000 ×  g for 5 min after the indicated treatments. Then, cells were re-suspended in 70% (v/v) cold ethanol and stored at − 20 °C overnight. After incubation at 37 °C with PI solution in the dark for 30 min, cell cycle distribution was analyzed by BD CellQuest Pro software by FACS Calibur.

### Molecular Docking

The structure of the human Src kinase domain in complex with cancer drug bosutinib was obtained from the Protein Data Bank (PDB code 4MXO). Crystal water and bosutinib were removed, and hydrogen atoms were added according to the protonation state of chemical groups at pH 7.0. The binding site was defined as a 4 Å radius around bosutinib. Isorhamnetin was then docked into the ATP binding site of the Src kinase domain using LeDock (http://lephar.com) on the basis of a combination of simulated annealing and evolutionary optimization of the ligand pose (position and orientation) and its rotatable bonds^28^.

### Kinase inhibition assays

Src kinase inhibition by different compounds was determined using Caliper Mobility Shift assays (Caliper Life Sciences, MA) according to the instructions provided^29^. Staurosporine was used as a reference compound.

### Western-Blotting

Whole cell protein extraction was conducted according to previous reports with slight modifications^13^. Protein samples (50 μ g) from each lysate were separated by SDS-PAGE and transferred to PVDF membranes (0.45 μ m), and blocked with 5% skimmed milk in Tris-buffered saline containing 0.1% Tween-20 (TBST) at room temperature for 1 h. The membranes were then incubated at 4 °C overnight with primary antibodies against pSrc^416^ (1:500), Src (1:1000), ATR (1:500), Wee 1 (1:500), CDK1 (1:8000), Cyclin B (1:500) and β -Actin (1:1000) and final incubation for 2 h in the dark with DyLightTM 680-labeled rabbit IgG (H+ L) antibody. The blots were developed with ECL Western Blotting Substrate (Thermo Fisher Scientific) and analyzed by scanning densitometry using a Tanon Image System (Tanon, China). β -Actin was used as an interval control. The prepared samples were separated by SDS-PAGE and transferred to PVDF membranes (0.45 μ m). Membranes were blocked with 5% skimmed milk and incubated overnight at 4 °C with the primary antibody before addition of the secondary antibody (1:1000 dilution) and final incubation for 2 h in the dark. The intensity of bands was measured with an Oddysey Fc. detection system (Li-cor).

### siRNA transfection

The Src siRNAs were designed and synthesized by GenePharma Co., Ltd (Shanghai, China). K562 cells were transiently transfected with siRNA (100 nmol/L) using Lipofectamine 3000 (Life Technologies) according to the manufacturer’s instructions. After transfected by siRNA for 48 h, K562 cells were collected and then treated by indicated experimental requirements.

### Statistical analysis

Statistical analysis was performed with the GraphPad Prism 5.0 software. All data were depicted as the mean ±  standard deviation of individual values from at least three independent experiments. Statistical variation was calculated with the Student’s t-test. Statistical significance was set at *p* <  0.05.

## Additional Information

**How to cite this article**: Wu, X. *et al.* Characterization of anti-leukemia components from *Indigo naturalis* using comprehensive two-dimensional K562/cell membrane chromatography and *in silico* target identification. *Sci. Rep.* **6**, 25491; doi: 10.1038/srep25491 (2016).

## Supplementary Material

Supplementary Information

Supplementary Table S1

Supplementary Table S2

Supplementary Table S3

## Figures and Tables

**Figure 1:**
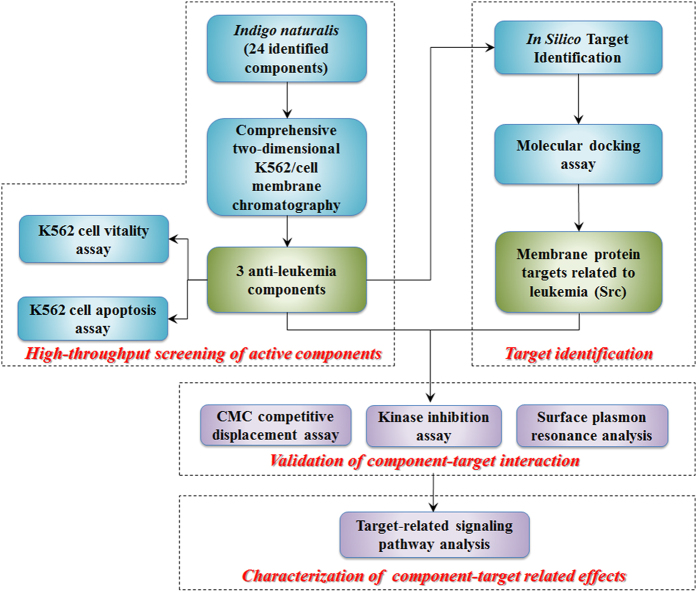
The flow diagram of characterizing anti-leukemia components and their targets from *Indigo naturalis* by the combination of comprehensive 2D K562/cell membrane chromatographic system and *in silico* target identification.

**Table 1:** Retention components identified by TOFMS.

Identification	tR(1^st^ K562/CMC, min)	tR(2^nd^ RPLC, min)	Fomula	Selected ion	Expected	Detected	Error (ppm)	Abund match(%)
indigo	2.5–7.5	9.47	C_16_H_10_N_2_O_2_	[M+ H]^+^	263.0815	263.0813	0.76	98.98
indirubin	22.5–37.5	9.47	C_16_H_10_N_2_O_2_	[M+ H]^+^	263.0815	263.0813	0.76	98.98
tryptanthrine	2.5–30	8.18	C_15_H_8_N_2_O_2_	[M+ H]^+^	249.0659	249.0664	− 2.06	99.53
isorhamnetin	5–27.5	9.35	C_16_H_12_O_7_	[M+ H]^+^	317.0656	317.0655	0.3	98.26

**Table 2:** Eight common targets obtained by PharmMapper server and TargetHunter ranked by fit score according to PharmMapper server docking results.

Rank	PDB ID	Target Name	Number of Feature	Fit Score	Normalized Fit Score	z’-score
5	1i5r	Estradiol 17-beta-dehydrogenase 1	16	4.64	0.29	1.603
10	2dux	Aldose reductase	12	4.441	0.3701	0.917026
11	2bdj	Proto-oncogene tyrosine-protein kinase Src	6	4.413	0.7354	2.79099
51	1r1h	Neprilysin	10	3.889	0.3889	− 0.495387
73	1l0g	Beta-lactamase	9	3.795	0.4216	− 0.250371
133	2p4j	Beta-secretase 1	13	3.687	0.2836	− 0.430497
189	1pl6	Sorbitol dehydrogenase	5	3.611	0.7222	− 0.617597
212	1v3i	Beta-amylase	8	3.585	0.4481	− 0.715818
