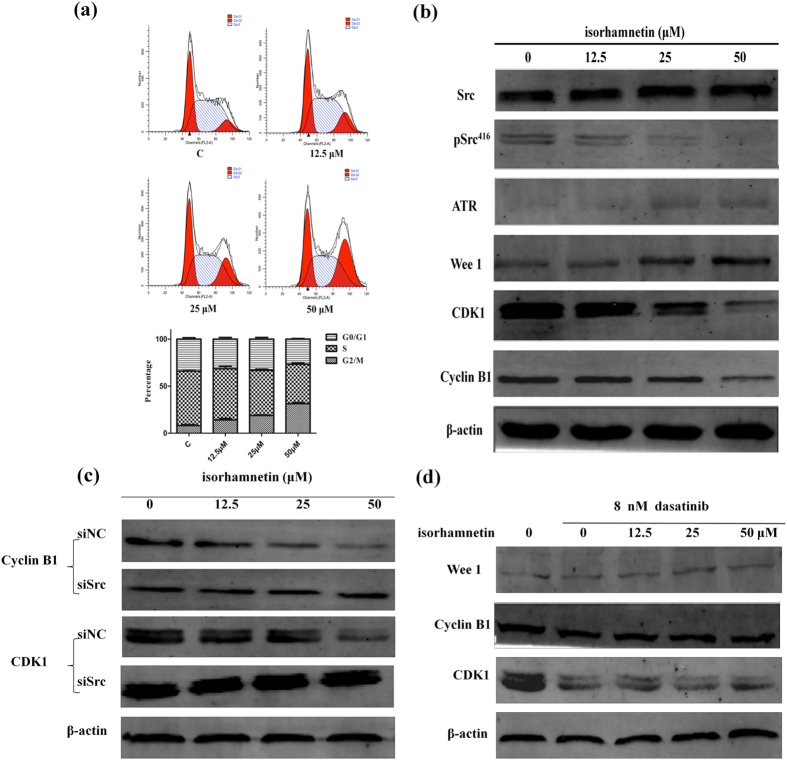# Corrigendum: Characterization of anti-leukemia components from *Indigo naturalis* using comprehensive two-dimensional K562/cell membrane chromatography and *in silico* target identification

**DOI:** 10.1038/srep30103

**Published:** 2016-08-26

**Authors:** Xunxun Wu, Xiaofei Chen, Dan Jia, Yan Cao, Shouhong Gao, Zhiying Guo, Philipp Zerbe, Yifeng Chai, Yong Diao, Lei Zhang

Scientific Reports 6: Article number: 2549110.1038/srep25491; published online: 05 06 2016; updated: 08 26 2016

The original version of this Article contained an error in the spelling of Dan Jia which was incorrectly given as Jia Dan. As a result, the Author Contribution statement now reads:

"X.F.C., Y.D. and L.Z. conceived and designed the experiments; X.X.W., D.J., Y.C., Z.Y.G. and S.H.G. performed the experiments; X.X.W., X.F.C. analyzed the data; X.X.W., X.F.C. wrote the manuscript; Y.F.C., P.Z. and Y.D. reviewed the manuscript."

In Figure 5c, the labels for the CDK1 panels were inverted, where ‘siSrc’ and ‘siNC’ were incorrectly given as ‘siNC’ and ‘siSrc’ respectively. The correct Figure 5 appears below as Figure 1.

These errors have now been corrected in the PDF and HTML versions of the Article.

## Figures and Tables

**Figure 1:**