# Supplementary material for: Characterization of anti-leukemia components from Indigo naturalis using comprehensive two-dimensional K562/cell membrane chromatography and in silico target identification
Source: Sci Rep. 2016 May 6;6:25491. doi: 10.1038/srep25491 (PMC4858665; doi:10.1038/srep25491)
Supplement: Supplementary Information [file srep25491-s1.doc]

**Supplementary information**

**Characterization of anti-leukemia components from *Indigo naturalis* using comprehensive two-dimensional K562/cell membrane chromatography and** ***in silico* target identification**

**Xunxun Wu^1,2,a^, Xiaofei Chen^2,a^, Dan Jia^2^, Yan Cao^2^, Shouhong Gao**^2^**, Zhiying Guo^1,2^, Philipp Zerbe^3^, Yifeng Chai^2^, Yong Diao^1*^, Lei Zhang^2*^**

^1^School of Biomedical Science, Institute of Molecular Medicine, Huaqiao University, Quanzhou 362021, PR China

^2^ School of Pharmacy, Shanghai Changzheng Hospital, Second Military Medical University, Shanghai 200433, PR China

^3^Department of Plant Biology, University of California, Davis, CA 95616, USA

**
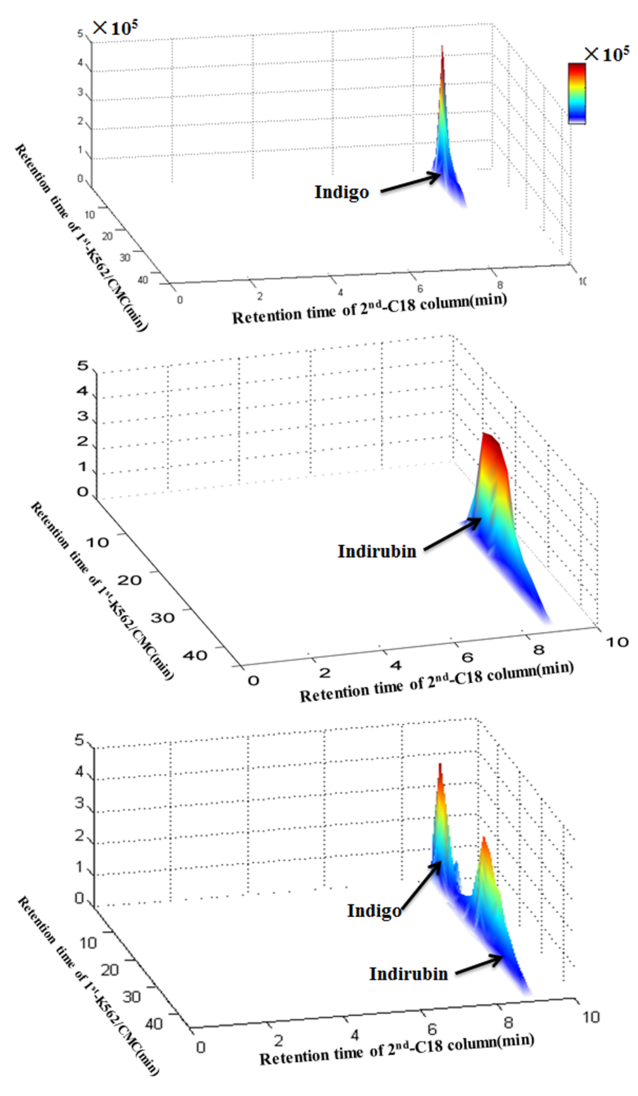
**

**Figure. S1** Three-dimensional plot of indirubin, indigo and their mixed standards obtained using the two-dimensional K562/CMC system.


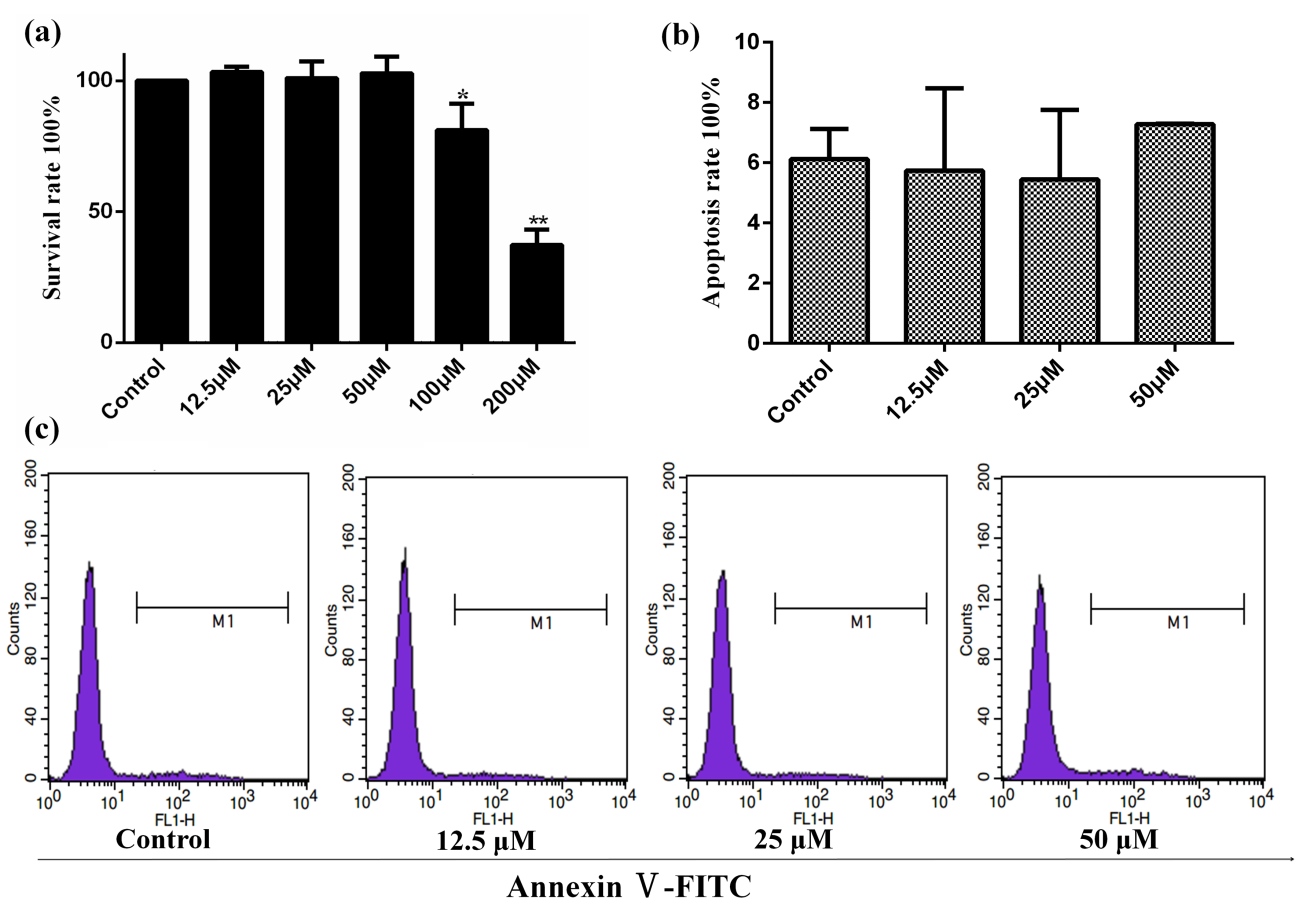


**Figure. S2 Cell vitality and apoptosis induced by indirubin.** (a) Effect of indirubin on cell viability in K562 cells. Cell vitality of indirubin on K562 cells measured by CCK-8 assay after exposed to increased concentrations (0-200 μM) for 48h. (b) and (c) Cell apoptosis of indirubin on K562 cells measured by Annexin V-FITC assay after exposed to increased concentrations (0-50 μM) for 48h. All data were shown as mean standard deviation (n=6). Statistical significance was calculated by Student’s t-test.


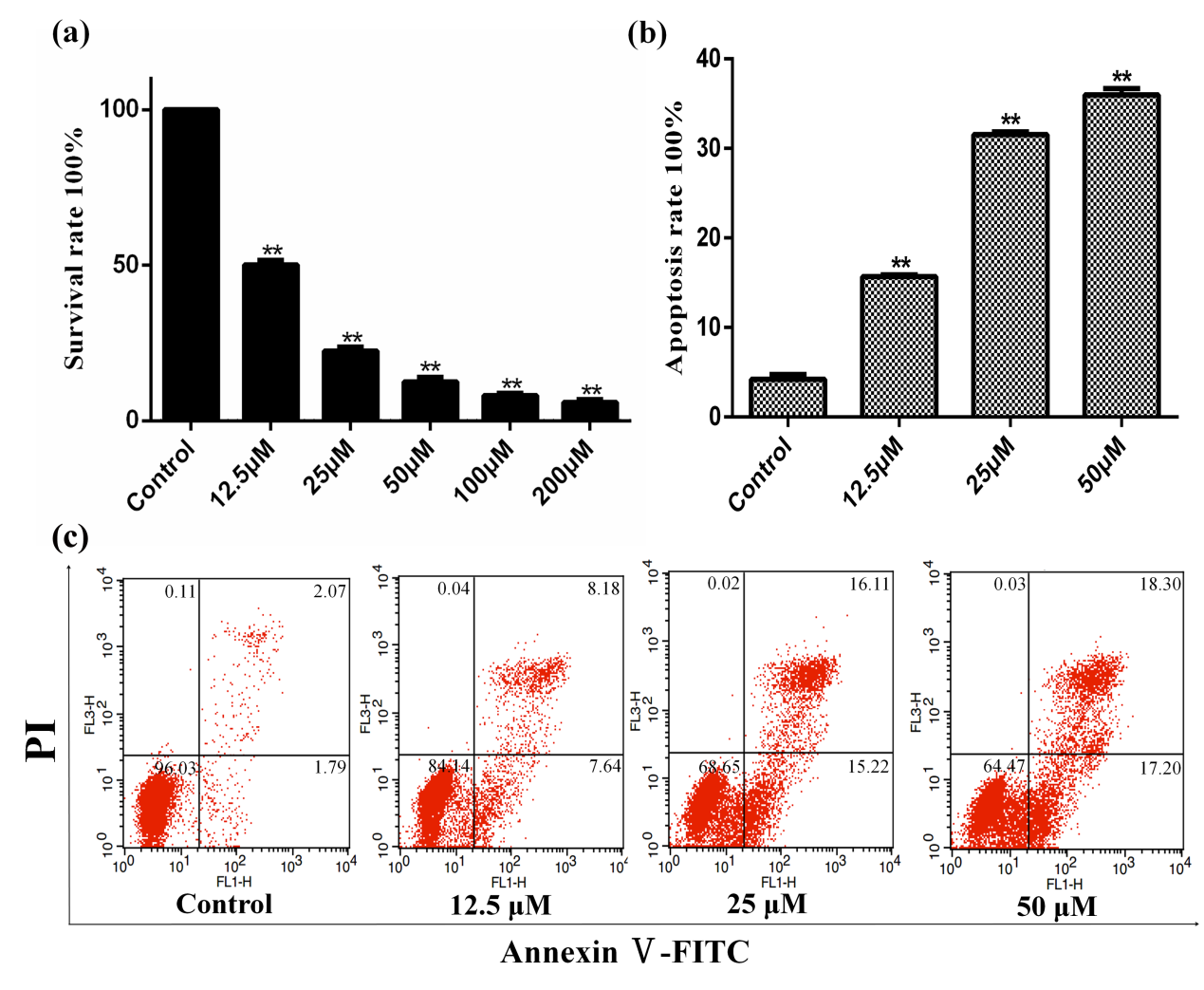


**Figure. S3** **Cell vitality and apoptosis induced by tryptanthrin.** (a) Effect of tryptanthrin on cell viability in K562 cells measured by CCK-8 assay after exposed to increased concentrations (0-200 μM) for 48h. (b) and (c) Cell apoptosis of tryptanthrin on K562 cells measured by Annexin V-FITC assay after exposed to increased concentrations (0-50 μM) for 48h. All data were shown as mean standard deviation (n=6). Statistical significance was calculated by Student’s t-test.


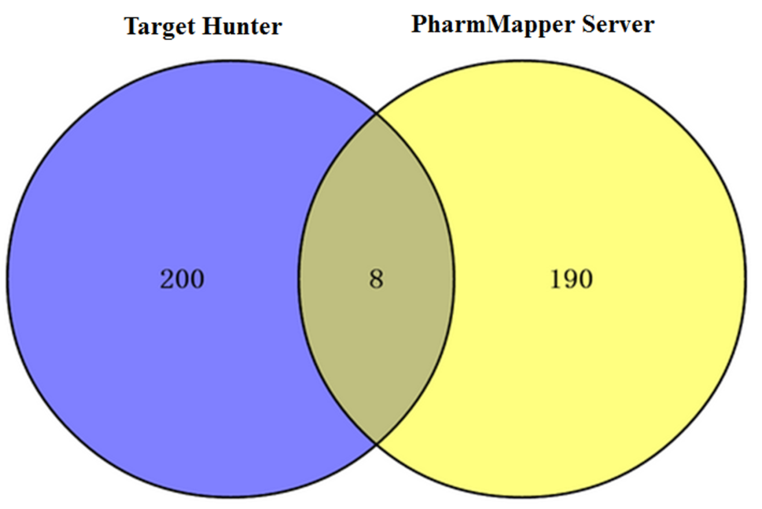


**Figure. S4** Analysis of common targets by Venny obtained from the PharmMapper Server and TargetHunter tools.


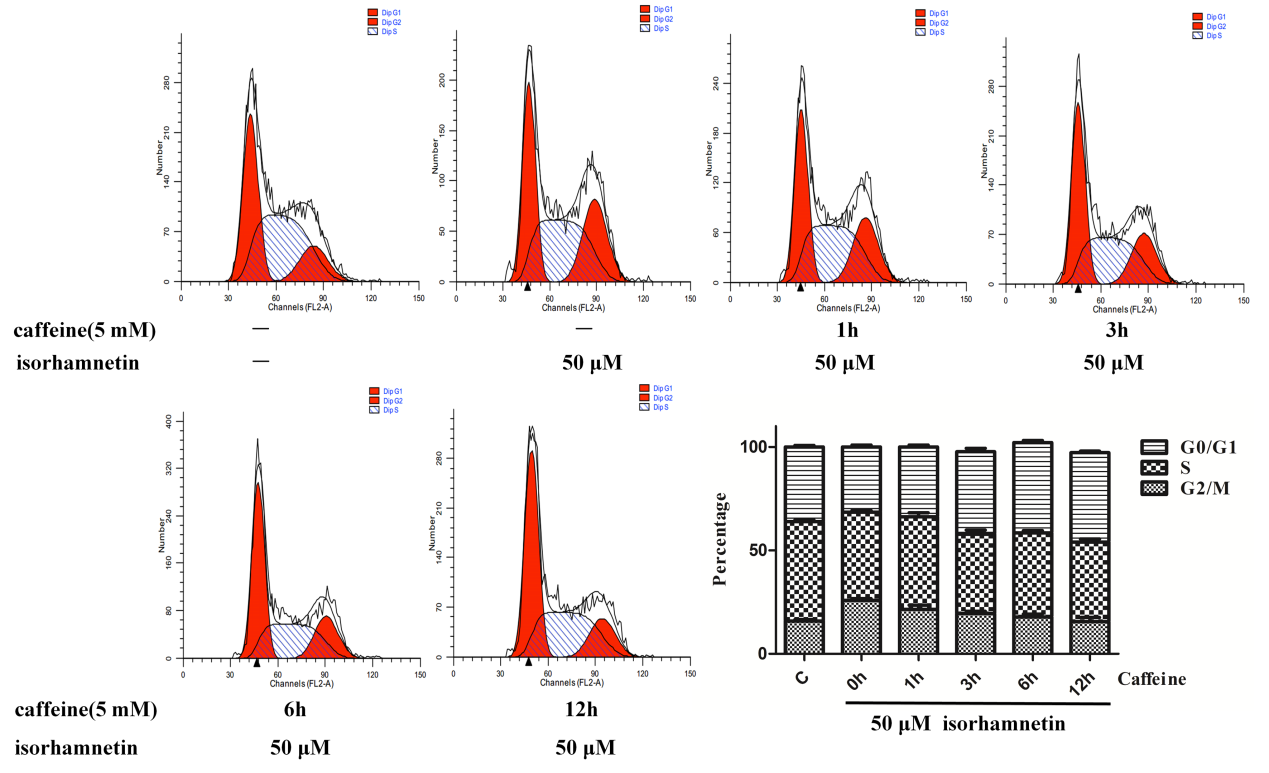


**Figure. S5** Flow cytometry analysis of the effects of caffeine (5 mM) on cell-cycle progression after 50 μM isorhamnetin pre-treated for 24h

**Table S4** Sequences of the siRNA used for the experiment.

| Name | Direction | Sequence |
| --- | --- | --- |
| Scrambled siRNA | Sense | 5’-UUCUCCGAACGUGUCACGUTT-3’ |
|  | Antisense | 5’-ACGUGACACGUUCGGAGAATT-3’ |
| Src siRNA-1 | Sense | 5’-GCUCCAGAUUGUCAACAACTT-3’ |
|  | Antisense | 5’-GUUGUUGACAAUCUGGAGCTT-3’ |
| Src siRNA-2 | Sense | 5’-GCGGUUACUGCUCAAUGCATT-3’ |
|  | Antisense | 5’-UGCAUUGAGCAGUAACCGCTT-3’ |
| Src siRNA-3 | Sense | 5’-CCUUCCUGGAGGACUACUUTT-3’ |
|  | Antisense | 5’-AAGUAGUCCUCCAGGAAGGTT-3’ |


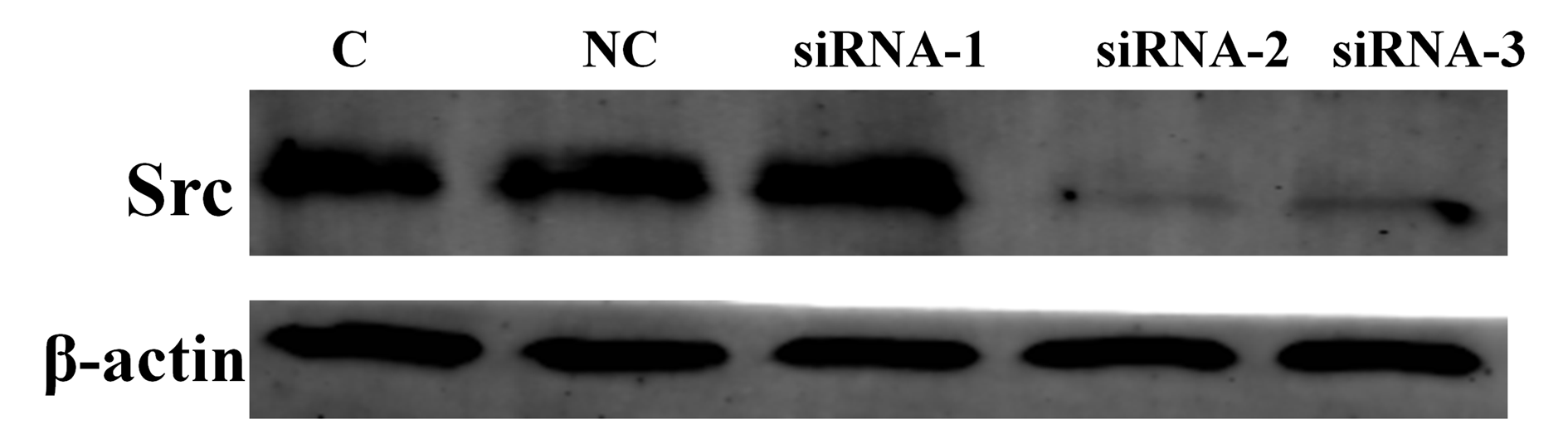


**Figure. S6** The effects of Src siRNA and negative control (NC) siRNA on K562 cells.

**
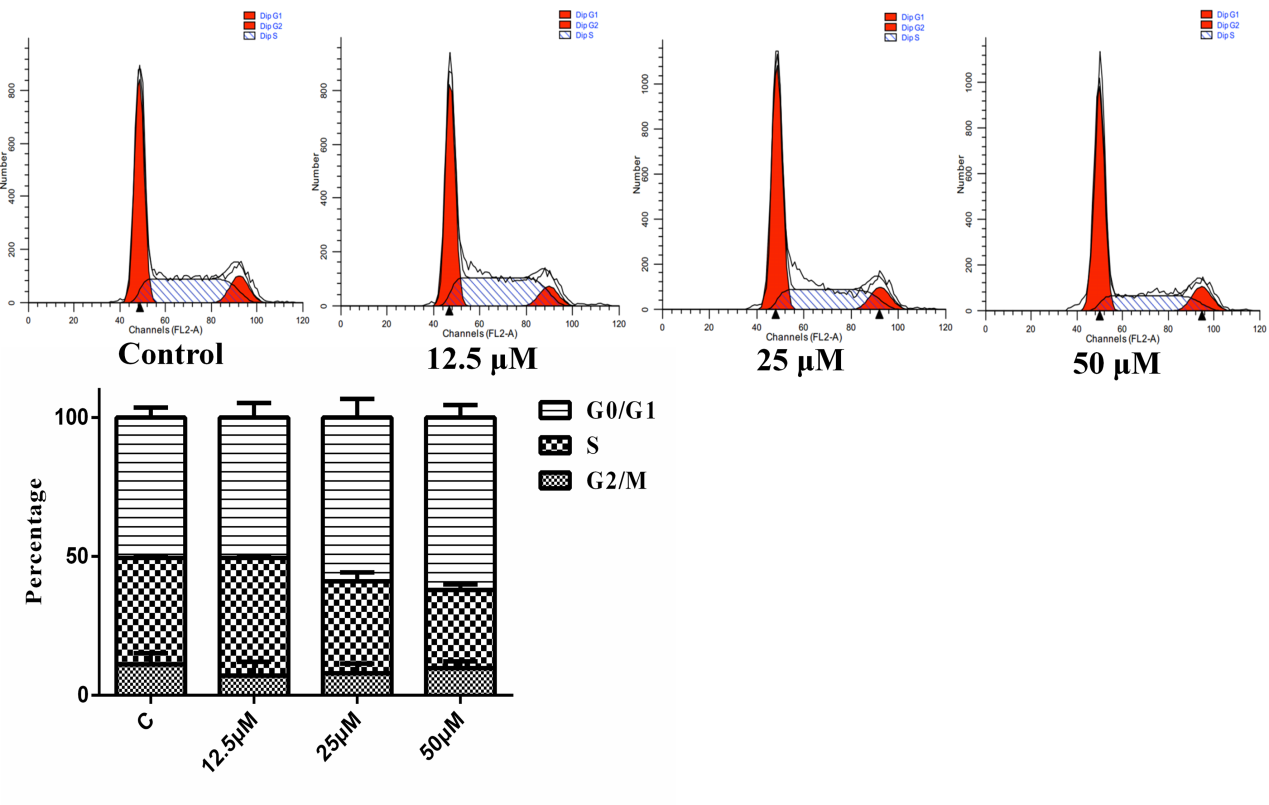
**

**Figure. S7** Cell cycle analysis of the K562 cells transfected with Src siRNA after treated by isorhamnetin (0-50 μM) for 24h.

**
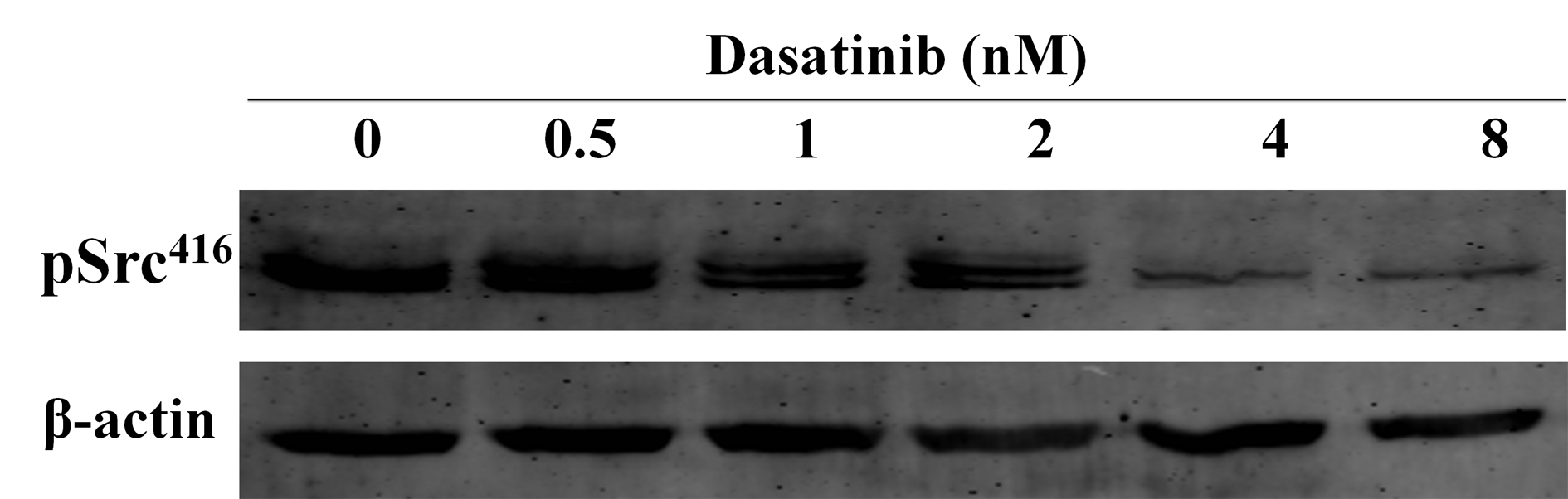
**

**Figure. S8** The phosphorylation levels of Src^416^ protein treated by different concentration (0-8 nM) of dasatinib for 12h.

**
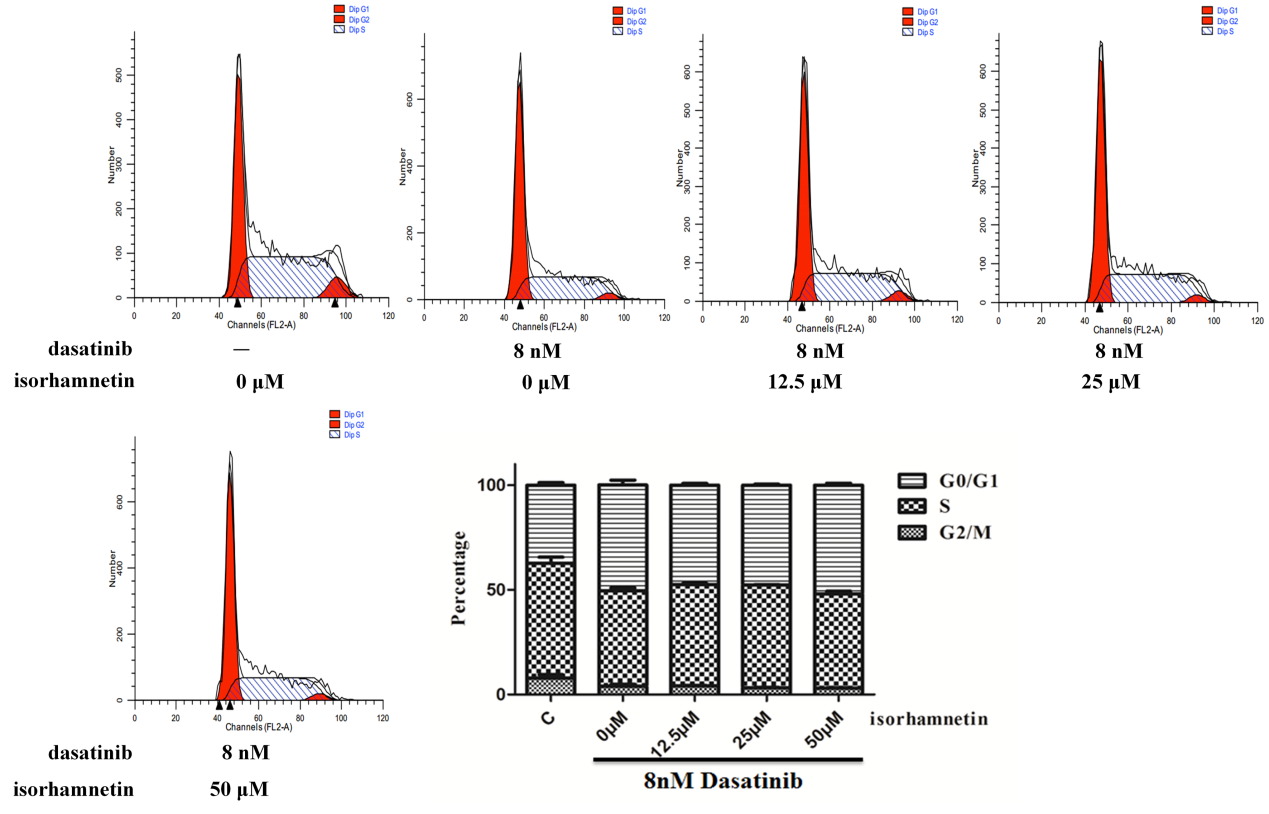
**

**Figure. S9** Flow cytometry analysis of the effects of isorhamnetin on cell-cycle progression after 8 nM dasatinib pre-treated for 12h. K562 cells were treated with isorhamnetin (0-50 μM) for 24h.

**Supplementary methods**

**Chromatographic conditions for Q-TOFMS**

Agilent 1290 UHPLC consisting of binary (Pump 2 and Pump 3) solvent delivery systems was adopted. Mobile phase A consisted of water with 0.1% formic acid (FA) and mobile phase B (acetonitrile containing 0.1% FA(v/v)) by a linear gradient elution program as follows: 0-2 min, 5% B; 2-17 min, from 5% B to 95% B; 17-19 min, 95% B; Two mobile phases A and B were used at flow rate of approximately 0.4 mL min^−1^, and then was introduced into G6538 Q-TOFMS (Agilent Technologies).

**Chromatographic conditions for TOFMS**

Agilent 1200 series HPLC system consisting of unitary (Pump 1) and binary (Pump 2 and Pump 3) solvent delivery systems. The mobile phases for 1^st^-k562/CMC column was 5 mM ammonia acetate, and the flow rate was 0.2 mL/min. The mobile phase for 2^nd^-C18 column was composed of solvent A (water with 0.1% formic acid (v/v)) and solvent B (acetonitrile with 0.1% FA (v/v)) by a linear gradient elution program as follows: For Qingdai extraction, 0-12 min, from 30% B to 85% B; 12-12.01 min, from 85% B to 30% B; 12.01-14 min, 30% B; for indigo, indirubin and their mixed standard solutions: 0-8 min, from 30% B to 85% B; 8-8.01 min, from 85% B to 30% B;8.01-10 min, 30% B; for imatinib and dexamethasone standard solutions: 0-4 min, from 30% B to 85% B; 4-4.01 min, from 85% B to 30% B; 4.01-5 min, 30% B. And then the eluent was split, and approximately 0.4 mL min^−1^ was introduced into 6220 TOF mass spectrometer (Agilent Technologies).

**Chromatographic condition for HPLC**

HPLC analysis was carried out on a Agilent 1200 liquid chromatography system (Agilent technologies Inc., Waldbronn, Germany) equipped with G1311A four pumps, a G1314A MWD UV detector and G1313A. Chromatographic conditions for the above systemS are as follows: the HPLC column was a Dimonsil C18 （5 mM, 250×4.6 mm）（Beijing， China）. Two mobile phases A and B were used at flow rate of 1 mL/min. Mobile phase A consisted of methyl alcohol with 0.2% formic acid (FA) and mobile phase B was water containing 0.2% FA. The proportion of mobile phase A and mobile phase B were 64:36. The column temperature was 25℃ and the injection volume was 10 μL.

**Mass spectrometer conditions for Q- TOFMS**

The Q-TOFMS (1290-G6538, analysis was performed using full scan mode and mass range was set at m/z 50–1100 in positive and negative ion mode. The conditions were as follows: drying gas (N2) flow rate, 11 L min^−1^; drying gas temperature, 350℃; Nebulizer, 45 psig; capillary voltage, 3500 V; fragmentor voltage, dynamic adjustment from 120 V; skimmer voltage, 60 V; and octopole RF, 250 V. All the data were processed by Agilent MassHunter Software Ver. B.07.00 (Agilent Technologies).

**Mass spectrometer conditions for TOFMS**

The 6220 TOFMS analysis was performed using full scan mode and mass range was set at m/z 110–1100 in positive ion mode. The conditions were as follows: drying gas (N2) flow rate, 10 L min−1; drying gas temperature, 350℃; Nebulizer, 40 psig; capillary voltage, 4000 V; fragmentor voltage, dynamic adjustment from 130 V; skimmer voltage, 60 V; and octopole RF, 250 V. All the data were processed by Agilent MassHunter Software Ver. B.07.00 (Agilent Technologies).

**Supplementary materials below are typical full blot of the western blot (M indicates marker):**

**1、K562 cells treated with 0-50μM isorhamnetin**


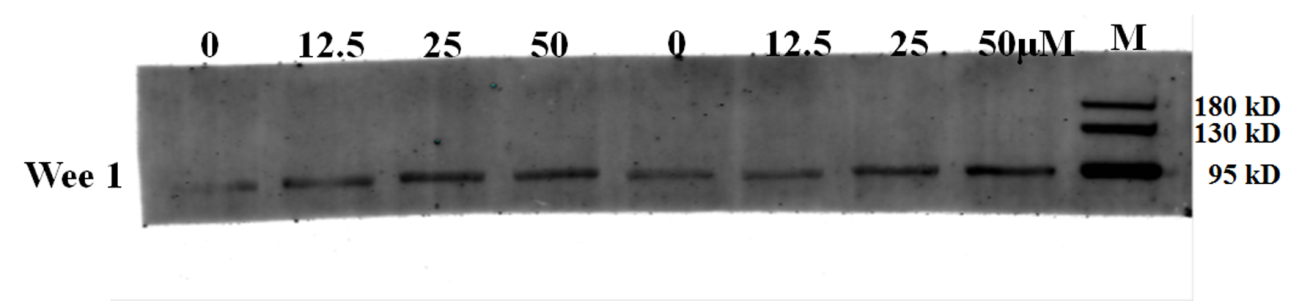


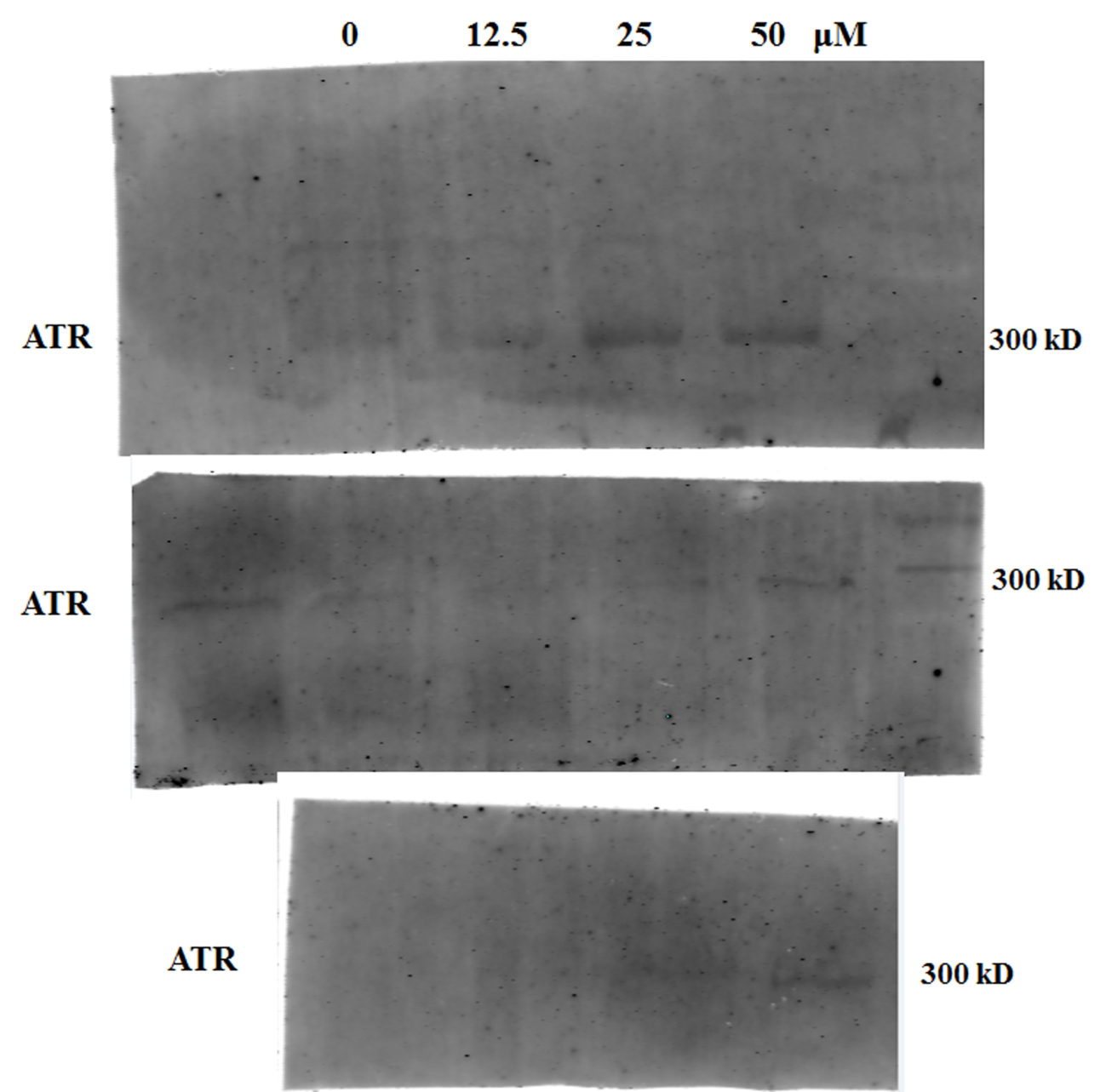


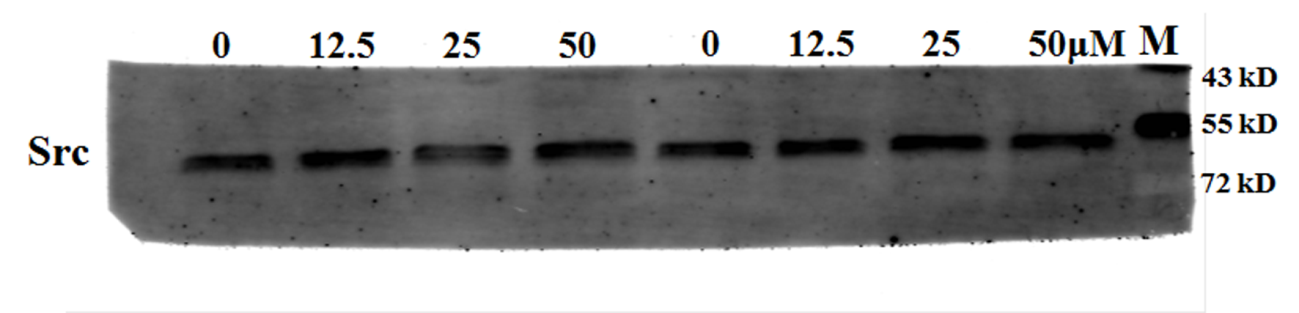

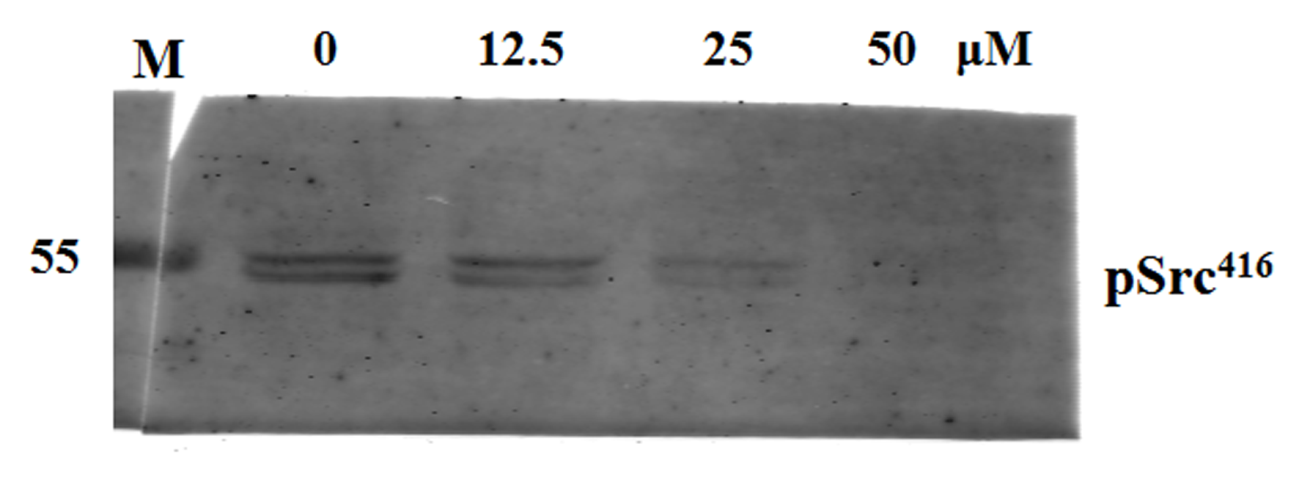


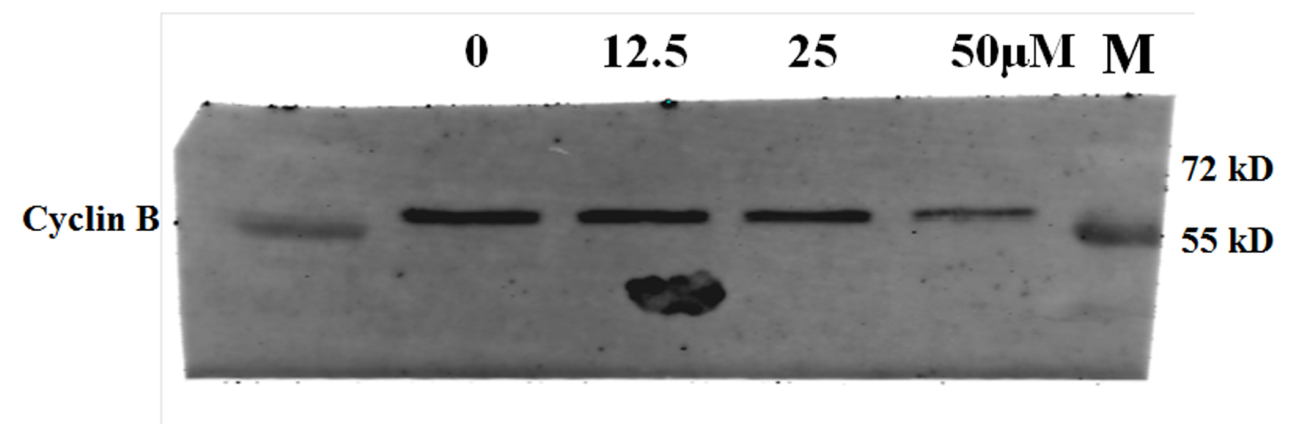


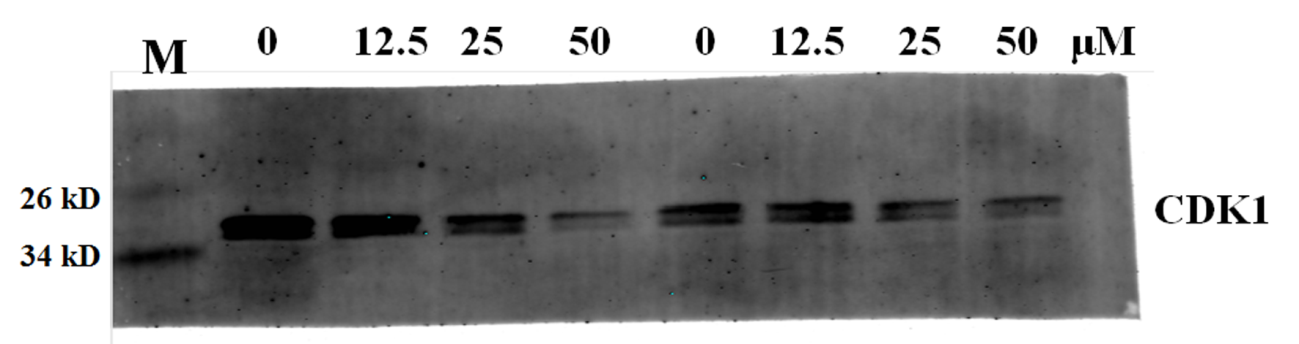


**2、phosphorylation state suppressed by dasatinib**

**
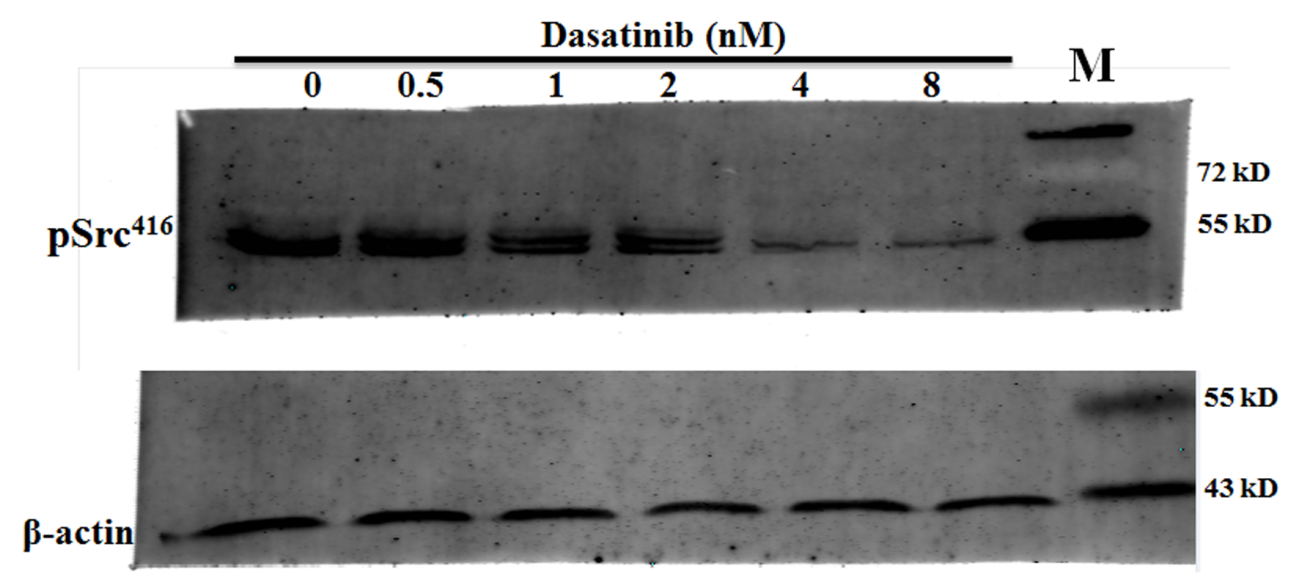
**

**
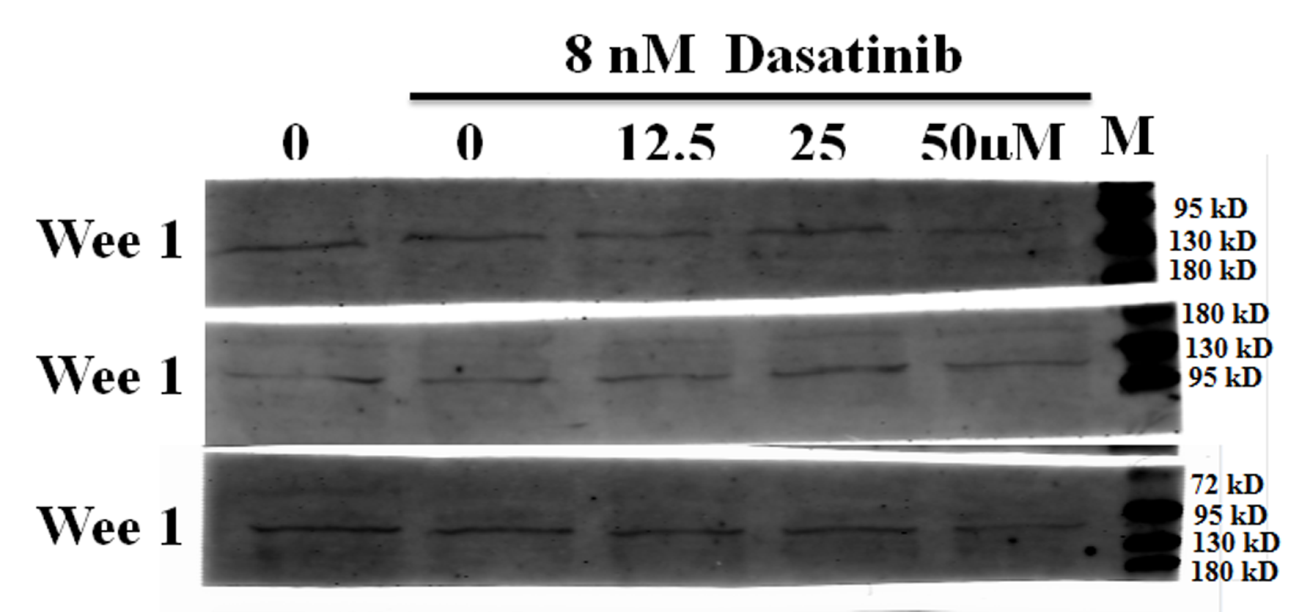
**

**
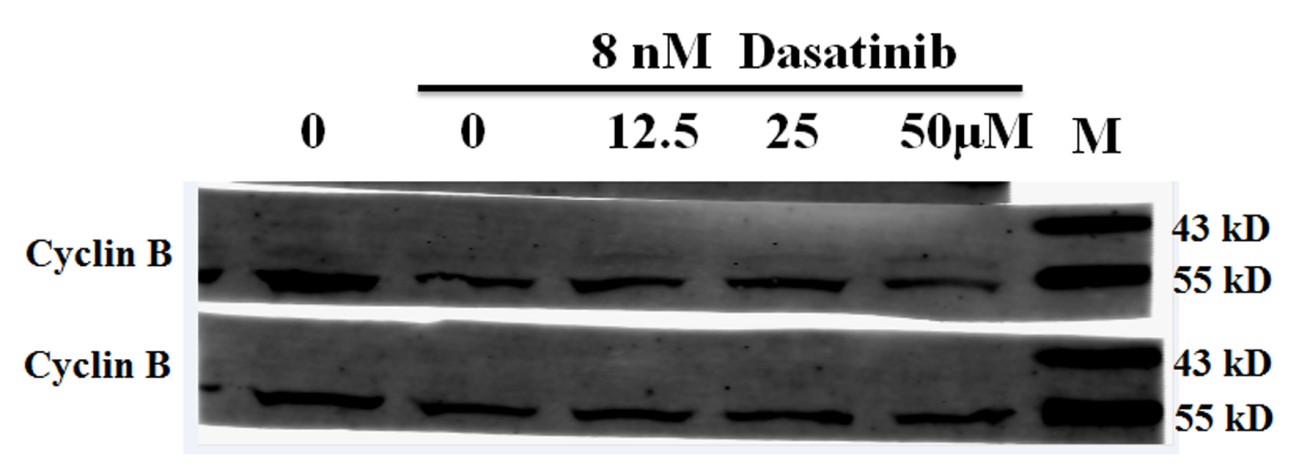
**

**
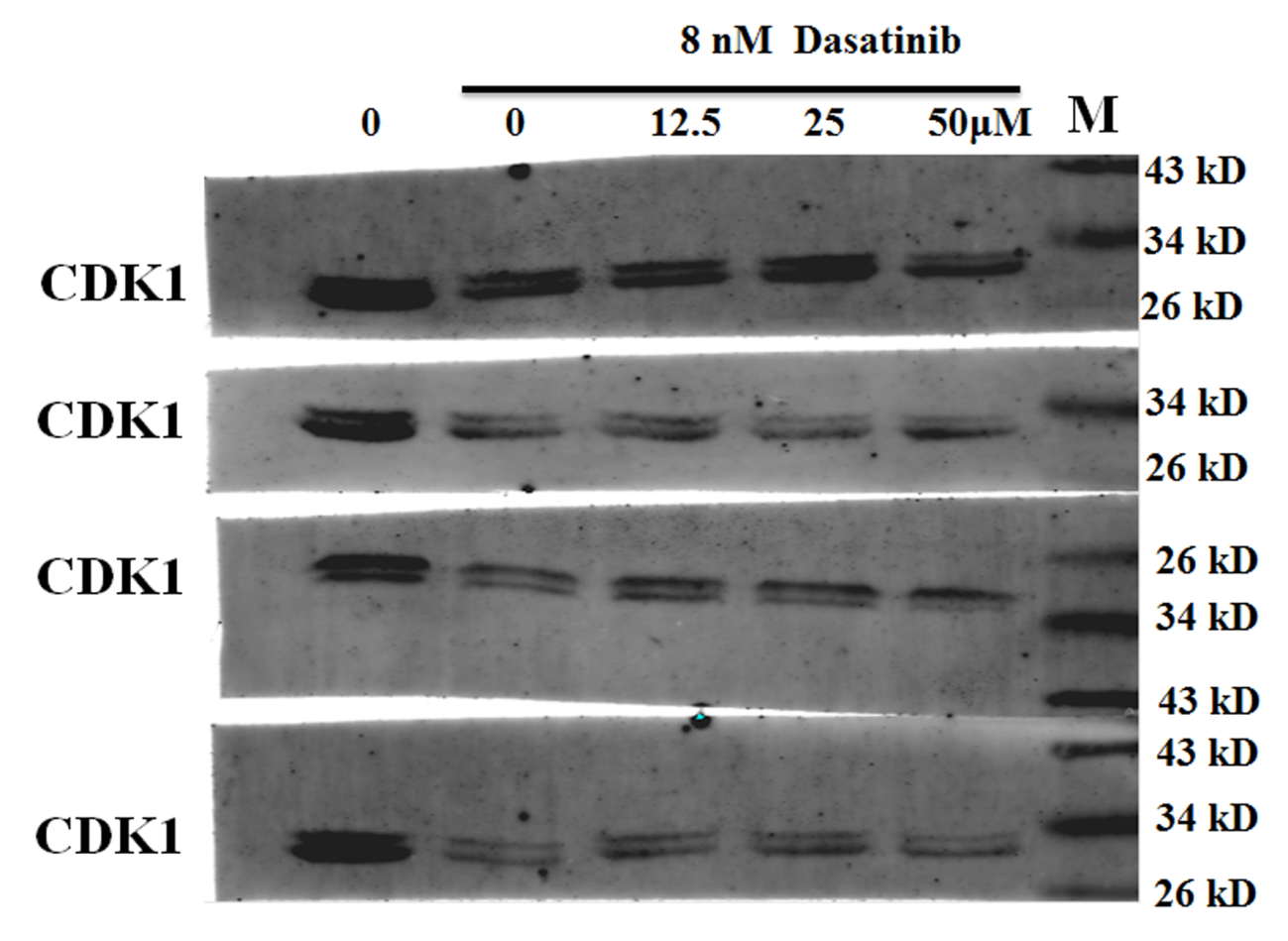
**

**
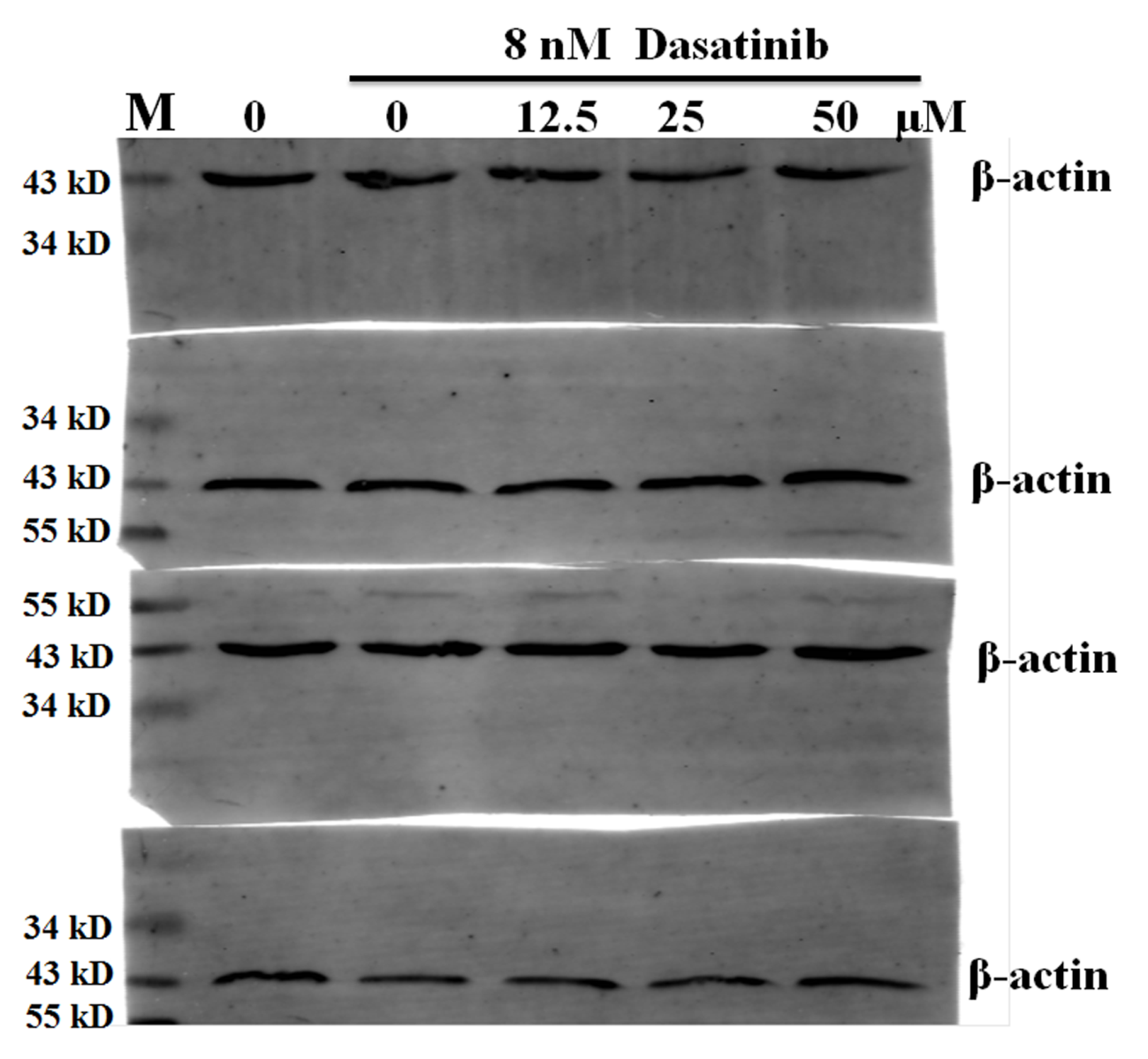
**

**3、Src receptor was knocked down with Src siRNA**

**
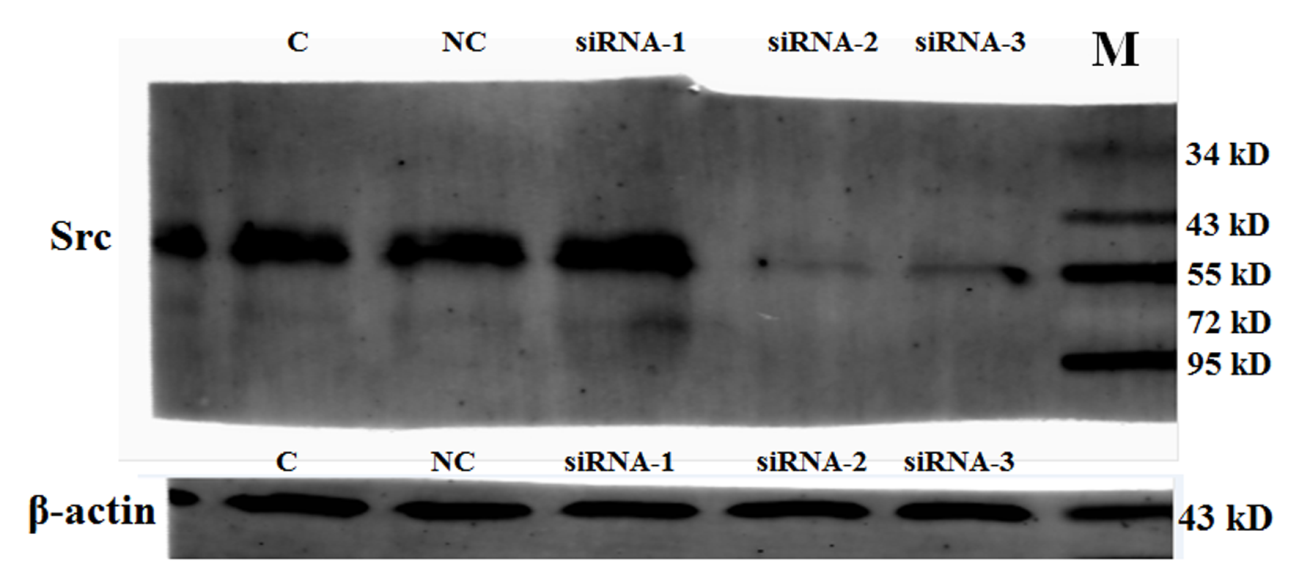
**

**
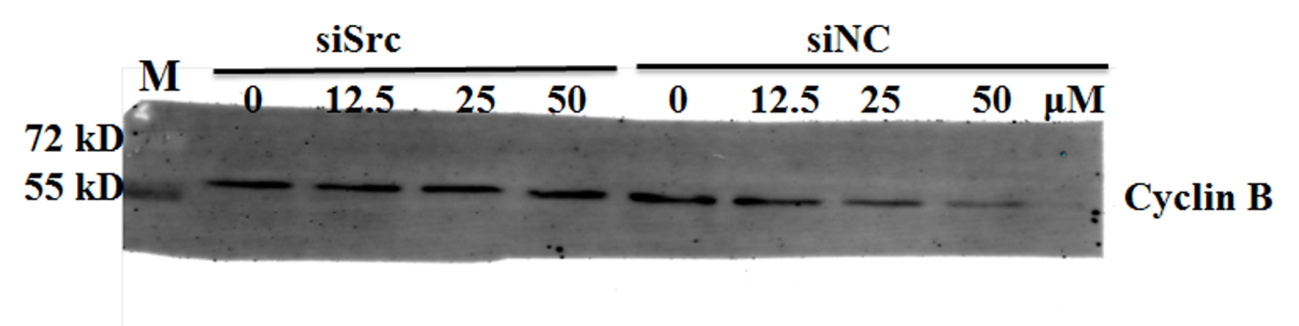
**

**
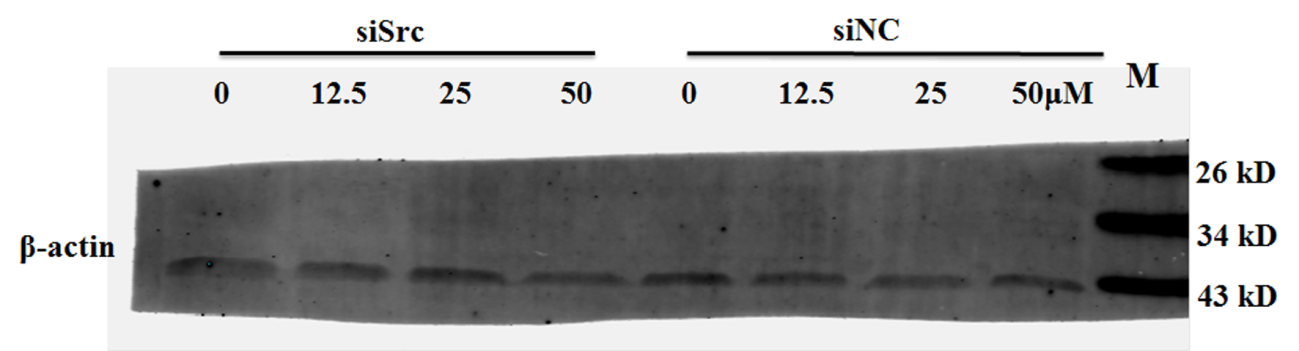
**

**
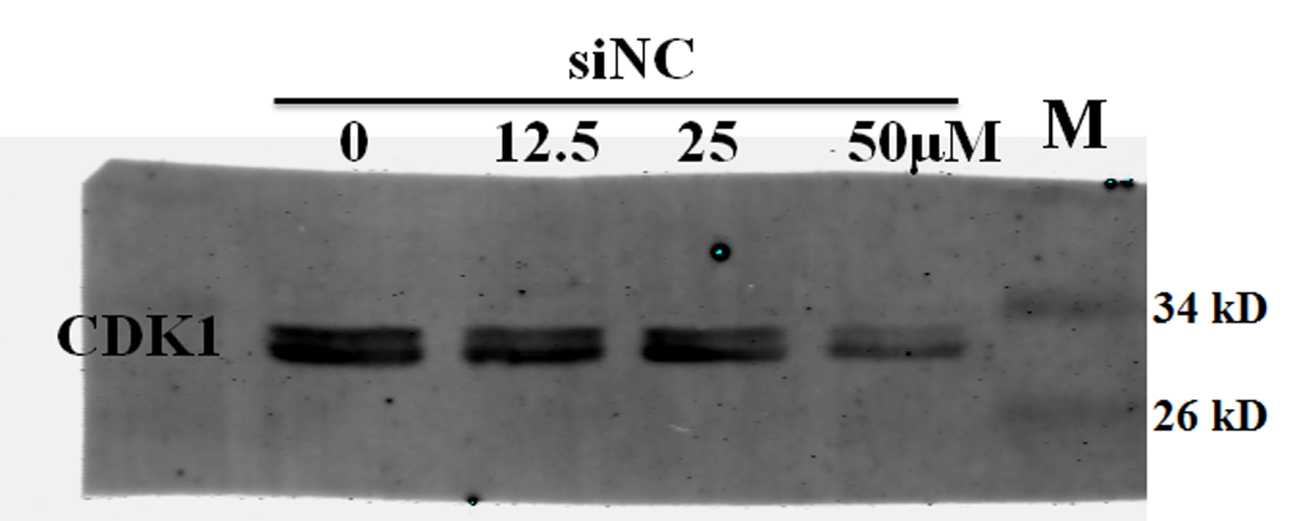

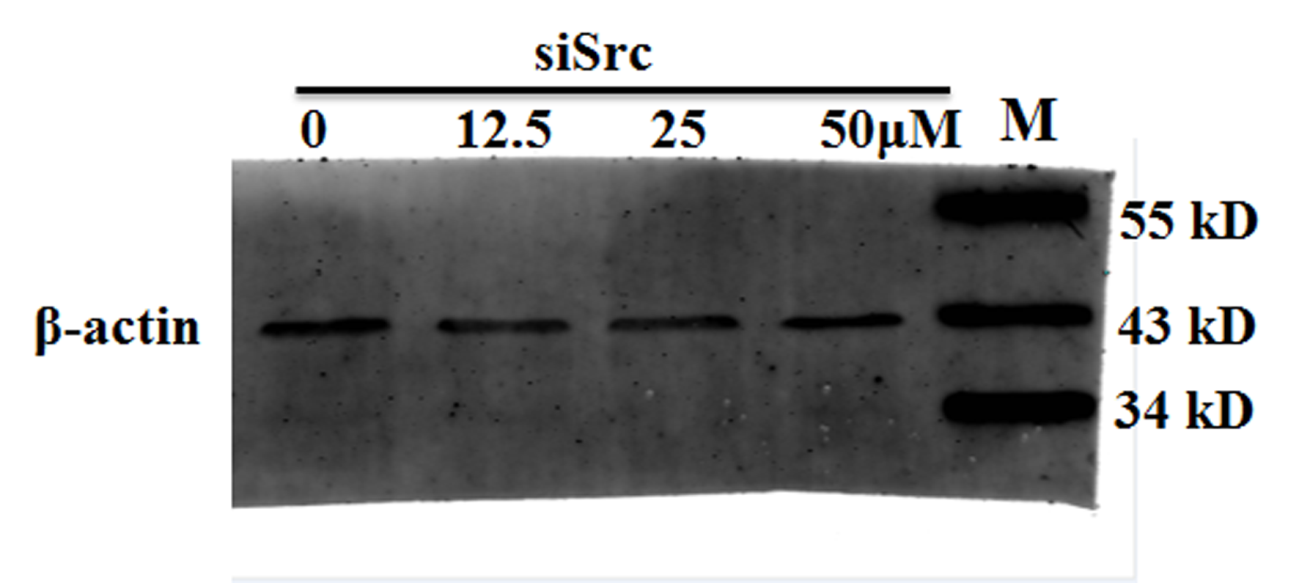
**

**
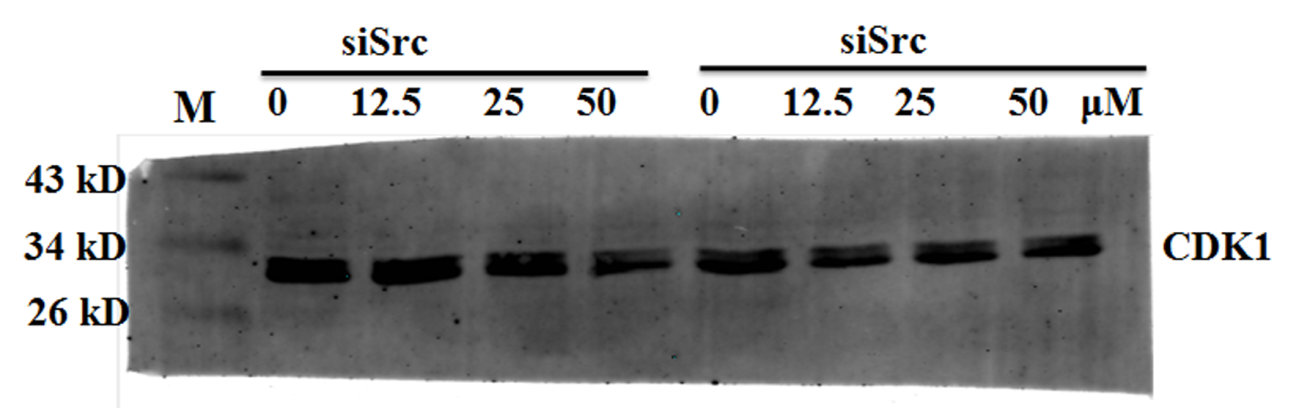

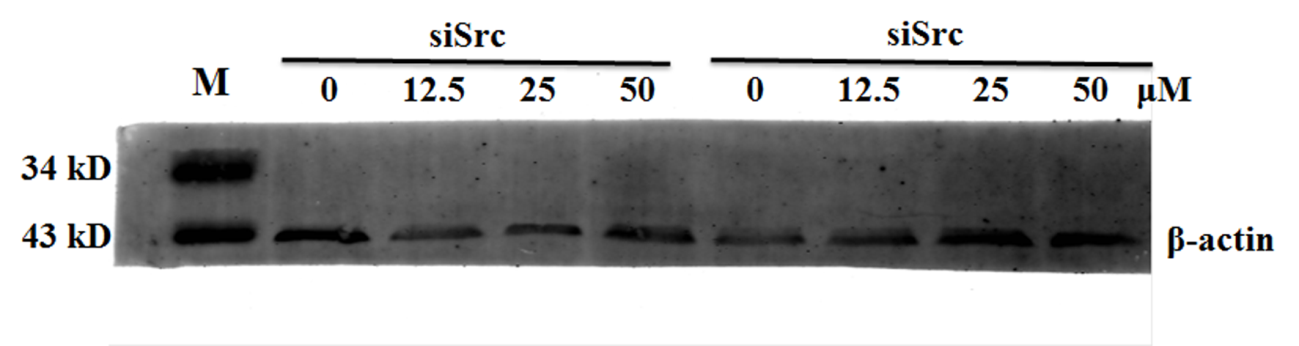
**
